# Functional Brachyury Binding Sites Establish a Temporal Read-out of Gene Expression in the *Ciona* Notochord

**DOI:** 10.1371/journal.pbio.1001697

**Published:** 2013-10-29

**Authors:** Lavanya Katikala, Hitoshi Aihara, Yale J. Passamaneck, Stefan Gazdoiu, Diana S. José-Edwards, Jamie E. Kugler, Izumi Oda-Ishii, Janice H. Imai, Yutaka Nibu, Anna Di Gregorio

**Affiliations:** Department of Cell and Developmental Biology, Weill Medical College of Cornell University, New York, New York, United States of America; The Wellcome Trust Sanger Institute, United Kingdom

## Abstract

The appearance of the notochord represented a milestone in Deuterostome evolution. The notochord is necessary for the development of the chordate body plan and for the formation of the vertebral column and numerous organs. It is known that the transcription factor Brachyury is required for notochord formation in all chordates, and that it controls transcription of a large number of target genes. However, studies of the structure of the *cis*-regulatory modules (CRMs) through which this control is exerted are complicated in vertebrates by the genomic complexity and the pan-mesodermal expression territory of *Brachyury*. We used the ascidian *Ciona*, in which the single-copy Brachyury is notochord-specific and CRMs are easily identifiable, to carry out a systematic characterization of Brachyury-downstream notochord CRMs. We found that *Ciona* Brachyury (Ci-Bra) controls most of its targets directly, through non-palindromic binding sites that function either synergistically or individually to activate early- and middle-onset genes, respectively, while late-onset target CRMs are controlled indirectly, via transcriptional intermediaries. These results illustrate how a transcriptional regulator can efficiently shape a shallow gene regulatory network into a multi-tiered transcriptional output, and provide insights into the mechanisms that establish temporal read-outs of gene expression in a fast-developing chordate embryo.

## Abstract 2

During notochord formation in chordate embryos, the transcription factor Brachyury employs different regulatory strategies to ensure the sequential activation of downstream genes and thereby the deployment of a specific developmental program at the right time and place.

## Author Summary

Transcription factors control where and when gene expression is switched on by binding to specific stretches of DNA known as *cis*-regulatory modules (CRMs). In this study, we investigated the architecture and composition of CRMs that direct gene expression in the notochord—a transient rod-like structure found in all embryos that belong to the phylum chordata, which includes humans. Here we used the sea squirt *Ciona*, a simple chordate, and analyzed how the transcription factor Brachyury ensures the appropriate deployment of its target genes at specific times during the sequential steps of notochord formation. We compared CRMs found in different notochord genes downstream of Brachyury, expecting to find genes associated with greater numbers of Brachyury binding sites to be expressed at higher levels. To our surprise, we found instead that a higher number of functional Brachyury binding sites is typical of CRMs associated with genes that are expressed early in notochord development, while single-site CRMs are characteristic of genes that are turned on during the intermediate stages of this process. Finally, CRMs associated with genes expressed late in notochord development do not contain functional Brachyury binding sites but are controlled by Brachyury indirectly, through the action of intermediary transcription factors. These differences explain how a transcription factor that is present at all stages in a certain cell type can generate a sequential transcriptional output of gene expression.

## Introduction

The transcription factor Brachyury plays a paramount role in mesoderm formation in animals with widely diverse body plans [1],[2]. In chordate embryos ranging from sea squirts to mice, Brachyury is required for the formation of the notochord from axial mesoderm [3],[4]. In addition to its prominent function in notochord development, Brachyury has recently been shown to induce epithelial-mesenchymal transition when over-expressed in human carcinoma cells [5] and it has been described as a causative agent of chordomas, human tumors of presumed notochordal origin [6],[7].

Brachyury is believed to exert its multifaceted role by controlling the transcription of a large number of downstream effectors [8]–[10]. This point is proven by studies in various systems, including ascidians, zebrafish, mouse, and chordoma cell lines, all showing that Brachyury binds hundreds of genomic loci [11]–[15]. Although genome-wide studies have added numerous candidates to the list of mesodermal genes whose activity is influenced by Brachyury, the specific *cis*-regulatory mechanisms through which this factor performs its crucial function in notochord formation are still in need of elucidation. This is mainly due to the fact that detailed studies of *cis*-regulatory modules (CRMs) are complicated in vertebrate model systems by a number of intrinsic experimental limitations, including the early pan-mesodermal expression of Brachyury, genomic complexity, scarce accessibility of the notochord, slow embryonic development, and laborious transgenic protocols. However, in the ascidian *Ciona intestinalis*, an invertebrate chordate, expression of the single-copy *Brachyury* (*Ci-Bra*) gene is restricted to notochord cells by the action of the transcriptional repressor *Ciona* Snail [16]. In addition, compared to other chordates, the *Ciona* model system is characterized by a compact, fully sequenced genome, a readily distinguishable notochord, fast development, and ease of transgenesis [17]–[20].

The specificity of *Ci-Bra* expression in notochord cells has provided a unique experimental advantage for the initial identification of over 50 validated *Ciona* genes controlled by this transcriptional activator [11],[21]–[25]. More recently, the number of potential Ci-Bra target genes has surged to over 2,000 following genome-wide studies of chromatin occupancy by this factor in early embryos [13]. These observations have led to the assumption that Ci-Bra presides over a “shallow” gene network and controls the majority of its targets directly. In support of this view, the notochord CRMs associated with two early-onset Ci-Bra downstream genes, *Ci-tropomyosin-like* (*Ci-trop*) and *Ci-leprecan*, have been found to be controlled by Ci-Bra directly, through non-palindromic binding sites that share the consensus sequence TNNCAC [21],[26]. Remarkably, however, even though transcripts for Ci-Bra appear in notochord cells from the 64-cell stage and persist throughout embryogenesis [27],[28], many of its *bona fide* target genes are sequentially activated at various developmental stages [22],[25],[29]. The sequential deployment of Ci-Bra targets is crucial to ensure that the morphogenetic steps that lead to notochord formation progress seamlessly. As a result, within ∼8 h, notochord cells transition smoothly through invagination, convergent extension, cell-shape changes, lumen matrix secretion, and tube formation [30].

We sought to uncover how Ci-Bra establishes this sequential transcriptional output, and to this aim we analyzed the architecture and functional requirements of the notochord CRMs associated with a representative suite of Ci-Bra downstream genes expressed at different stages of notochord development. Upon completion of these analyses, the newly discovered *cis*-regulatory mechanisms were used to identify notochord CRMs from still uncharacterized Ci-Bra downstream genes and to predict their temporal onset of expression. The *in vivo* occupancy of the CRMs directly controlled by Ci-Bra was assessed by chromatin immunoprecipitation (ChIP) assays. This investigation yielded an evolutionarily conserved consensus sequence shared by functional Brachyury binding sites, led to a classification of the direct Ci-Bra target CRMs into different groups, and uncovered a relay mechanism that ensures the activation of late-onset Ci-Bra targets. We propose that these *cis*-regulatory strategies concertedly create a differential temporal read-out of the steady transcriptional input provided by Brachyury.

## Results

### Sequential Activation of *Bona Fide* Brachyury Target Genes in the Developing *Ciona* Notochord

**Figure 1:**
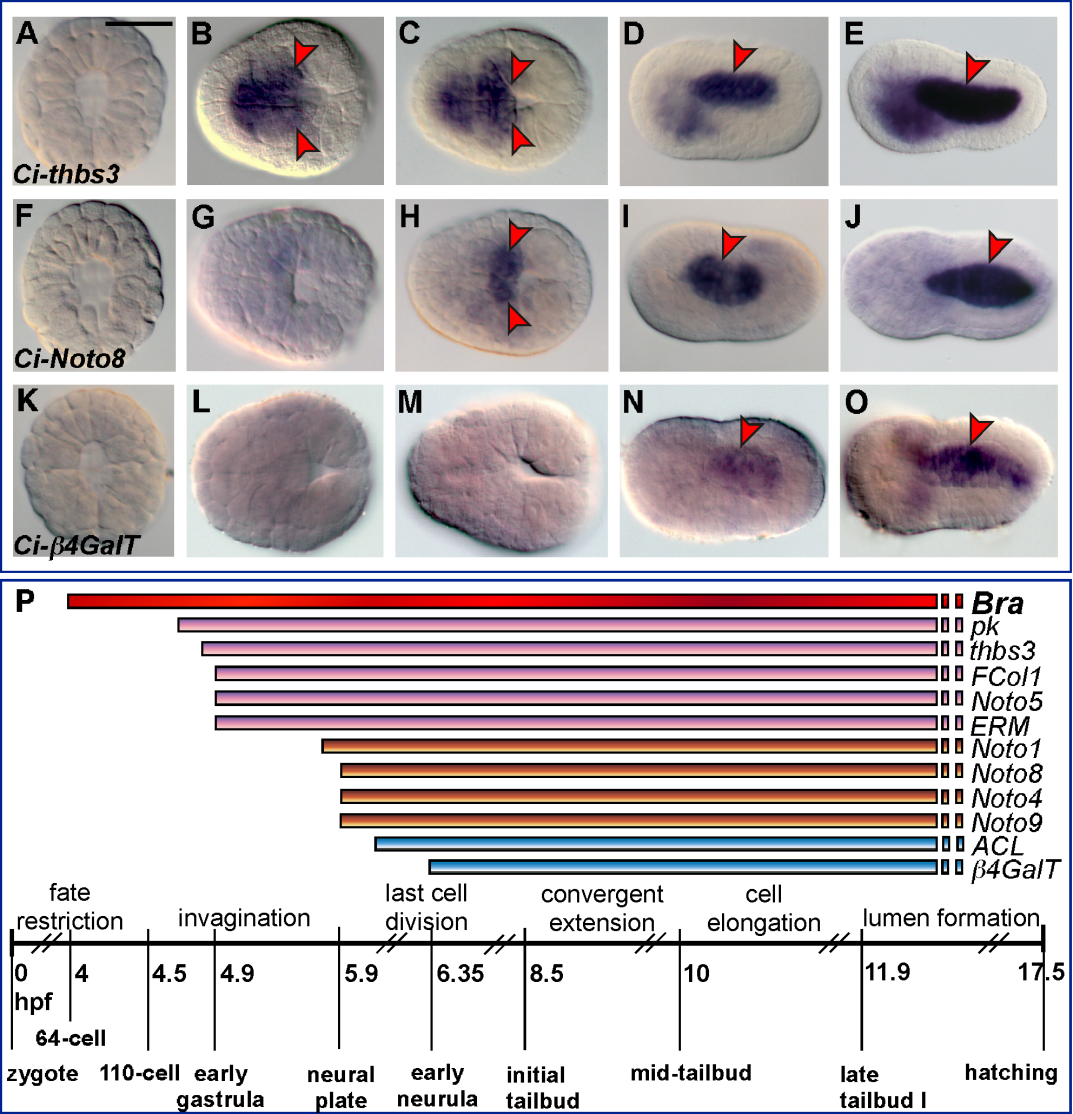
Sequential activation of Brachyury target genes in the developing notochord of *C. intestinalis*. (A–O) WMISH of *C. intestinalis* embryos at the 110-cell (A,F,K), gastrula (B,G,L), neural plate (C,H,M), neurula (D,I,N), and initial tailbud (E,J,O) stage. Embryos in (A,F,K) are photographed with anterior up, all other embryos are oriented with anterior to the left. Scale bar: approximately 100 µm. Red arrowheads indicate the regions containing stained notochord cells. (P) Schematic representation of the developmental time-courses of Ci-Bra (red horizontal bar) and its target genes (colored bars), plotted against a time-table denoting notochord morphogenetic events and the embryonic stages for *Ciona* at 18°C [32]. Early-onset genes are depicted as pink bars, middle-onset genes as orange bars, and the two late-onset genes are indicated by blue bars. All bars are dashed on their right side because the expression of the genes that they represent is yet to be determined at the larval stage. hpf, hours post-fertilization.

The expression patterns of numerous notochord genes that are likely controlled by Ci-Bra have been described in previous studies [11],[22],[23],[25],[31]. In this study we used whole-mount *in situ* hybridization (WMISH) to precisely determine the onset of notochord gene expression for a subset of *bona fide* Ci-Bra target genes for which this information was missing or incomplete (Figures 1A–1O and S1). The results of this analysis and of previous reports are summarized in Figure 1P and plotted against a time-course of the main developmental events that punctuate notochord formation in *Ciona*. From these comparisons, it is evident that the genes controlled by Ci-Bra fall within different classes, which we define here as early, middle, and late onset. Early-onset genes are detected in notochord precursors from gastrulation and include *Ci-prickle* (*Ci-pk*), the gene with the earliest onset [22], *Ci-thrombospondin3* (*Ci-thbs3*) (Figure 1A–1E), *Ci-fibrillar collagen* (*Ci-FCol1*) (Figure S1A–S1D), *Ci-Noto5* (Figure S1E–S1H), and *Ci-ezrin-radixin-moesin* (*Ci-ERM*) (Figure S1I–S1L). Middle-onset genes begin to be expressed in the notochord by late gastrulation, when the neural plate becomes distinguishable and is composed of ∼six rows of cells abutting the notochord precursors [32]; these genes include *Ci-Noto1* [22], *Ci-Noto8* (Figure 1F–1J), *Ci-Noto4* (Figure S1M–S1P), and *Ci-Noto9* (Figure S1Q–S1T). The late-onset genes include *Ci-ATP citrate lyase* (*Ci-ACL*), which is first detected at the late neural plate stage ([22] and our unpublished results) and *Ci-β1,4-Galactosyltransferase* (*Ci-β4GalT*), which is first detected at the neurula stage (Figure 1K–1O).

### Identification of Notochord CRMs Associated with Representative Ci-Bra Targets

We aimed at identifying the *cis*-regulatory mechanisms responsible for the differences observed in the developmental onset among Ci-Bra-downstream genes. To accomplish this goal, we employed a position-biased cloning strategy to identify notochord CRMs located within the genomic loci of *bona fide* Ci-Bra transcriptional targets representative of the early, middle and late-onset groups. Genomic fragments ranging from 1 to 3.7 kb were PCR-amplified from the 5′-flanking regions of 17 Ci-Bra target genes (Figures S2 and S3), accounting for a total of ∼43 kb of *C. intestinalis* genomic DNA. The size and genomic location of the initial fragments were selected on the basis of previously published work, which has shown that *Ciona* CRMs are compact sequences that frequently lie either in the 5′-flanking region or in the first intron of the gene with which they are associated [33].

Each fragment was cloned into the pFBΔSP6 vector upstream of the *Ci-FoxA-a* basal promoter region fused to *LacZ* [24] and tested *in vivo* for *cis*-regulatory activity by electroporation in *Ciona* zygotes [34]. Ten of the 17 genomic fragments were able to activate gene expression in notochord cells; of these, five, *Ci-Noto1*, *Ci-Noto8*, *Ci-Noto9*, *Ci-β4GalT*, and *Ci-ERM*, map to larger genomic regions that have been reported to display notochord activity by a parallel study from another group [35],[36]. In the majority of cases, the CRMs that we identified also directed expression in other tissues in addition to the notochord (Figure S2). Of the remaining seven genomic regions, five, two of which from the same locus (*Ci-Noto3*), were found to contain CRMs active in tissues other than the notochord; the patterns of activity exhibited by some of these fragments partially recapitulated the expression of their neighboring genes (Figure S3). In sum, of the 17 loci that were surveyed, eight harbored a notochord CRM near their 5′-end. In addition to these, one gene, *Ci-Noto5*, was found to contain a notochord CRM within its coding region, ∼9 kb downstream of its transcription start site, while the minimal functional sequences for *Ci-ACL* were found within its third intron.

### Ci-Bra Directly Controls a Subset of Notochord CRMs through Multiple Binding Sites

The characterization of minimal notochord CRMs was achieved through the analysis of sequence-unbiased serial truncations of the initial genomic fragments. Here we define as “minimal CRMs” those enhancer sequences, usually ranging from 65 to ∼300 bp, that are still able to direct a consistent, clearly detectable notochord staining *in vivo*. Once identified, the minimal CRMs were subjected to sequence-biased (i.e., binding site-specific) individual and combined point-mutation analyses. Putative binding sites were identified by scanning the sequences of each CRM with previously published consensus binding sites for *Ciona* notochord transcription factors, as well as using available databases. In particular, we used the consensus sequence TNNCAC to identify putative Ci-Bra binding sites, on the basis of previously published observations in *Ciona* [21] as well as in other chordates [37],[38].

We first attempted the dissection of the *Ci-pk* upstream region (Figure S2). We found that the 1.2-kb *Ci-pk* notochord CRM that we had identified is enriched in putative Ci-Bra binding sites with the generic TNNCAC core sequence (15 versus <5 expected by random occurrence). However, this notochord CRM relies not only upon the numerous putative Ci-Bra binding sites that are found in its distal region, but also on additional sequences located in its proximal region (unpublished data). This structural feature prevented the isolation and further dissection of minimal sequences required for the function of this CRM.

**Figure 2:**
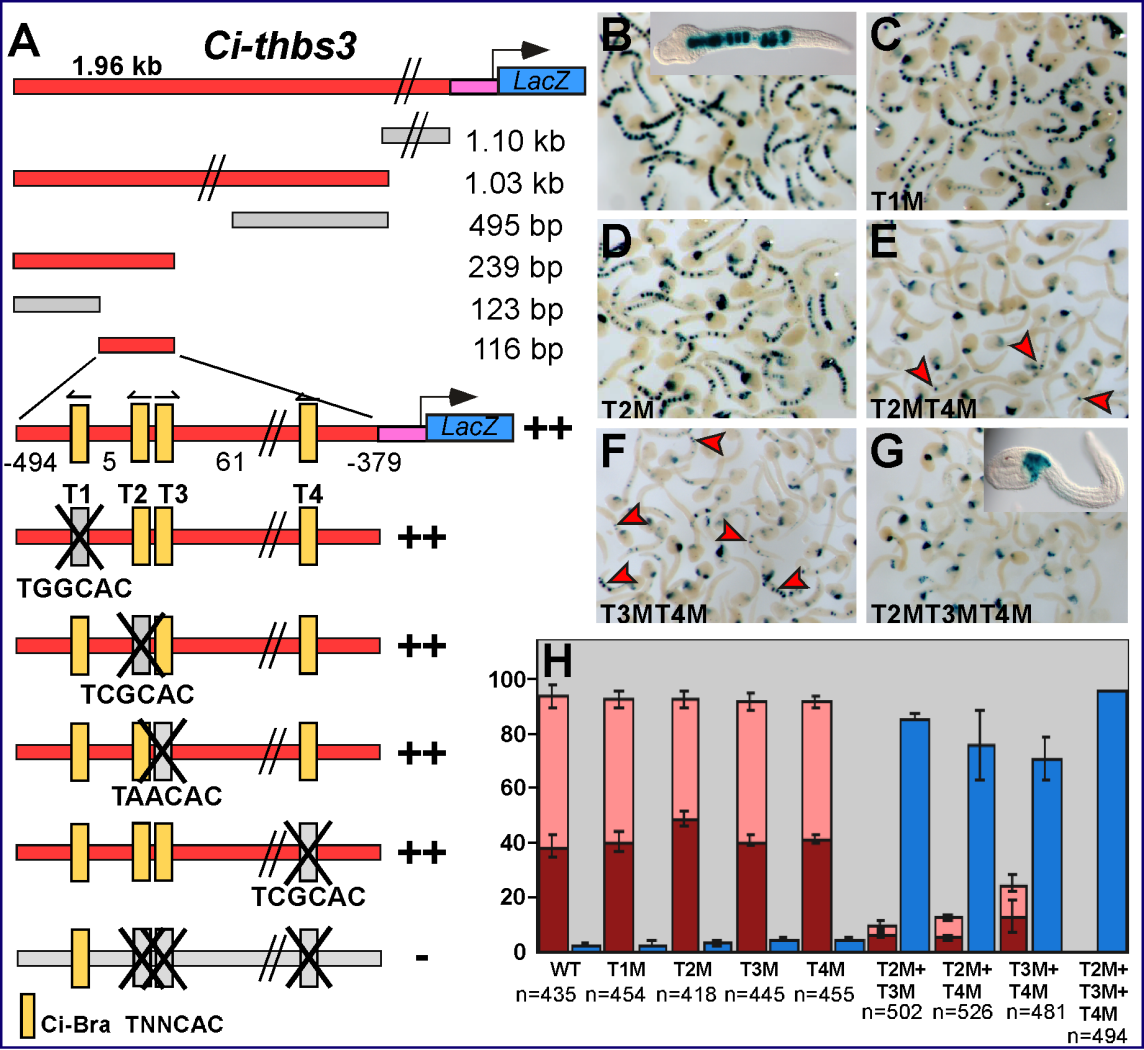
Synergistic activity of three Ci-Bra binding sites in the *Ci-thrombospondin 3* notochord CRM. Identification of the minimal sequences necessary for notochord activity of the *Ci-thbs3* notochord CRM through sequence-unbiased truncations and site-directed mutations. Red and grey rectangles symbolize genomic fragments displaying or lacking notochord activity, respectively. A pink rectangle indicates the *Ci-FoxA-a* basal promoter and a blue rectangle the *LacZ* reporter, both present in the electroporation vector (not drawn to scale; omitted in the truncation and mutation constructs for clarity). Parallel diagonal lines indicate genomic regions that are present in the constructs but are not depicted in the figure. “+” and “−” signs are used to show presence or absence of notochord activity, respectively. Putative binding sites are indicated by the symbols in the figure; their orientation is provided by small arrows on top of each site. Mutagenized sequences are depicted in grey and marked by an “X”. (A) Sequence-unbiased truncations and site-directed mutations. Putative Ci-Bra binding sites are indicated as T1–T4. (B–G) Low-magnification group microphotographs of late tailbud embryos electroporated in parallel with either the wild-type 116-bp *Ci-thbs3* notochord CRM (B) or with mutated versions of this construct carrying either individual (C,D), double (E,F), or triple (G) mutations in the Ci-Bra binding sites. The mutations were as follows (mutated sequences are in lower case): T1M, TGGCAC to TGGtct; T2M, TCGCAC to TCGtct; T3M, TAACAC to TAAtct; T4M, TCGCAC to TCGtct. Insets in (B,G) show higher magnification views of representative stained embryos. (H) Quantification of the activity of the constructs shown in (B–G) in different tissues, as detected by X-Gal staining. The *y*-axis reports the percentage of embryos showing staining in a certain tissue as a fraction of the total number of stained embryos scored. Dark red, activity in notochord only; pink, activity in notochord and in additional tissues; blue, activity only in tissues other than notochord. The number of stained embryos scored (*n*) for each construct is shown below the *x*-axis. Results were averaged from three independent experiments carried out on different batches of embryos.

In the case of *Ci-thrombospondin 3* (*Ci-thbs3*) [25], through serial truncations, we were able to reduce the original 1.96-kb genomic region (Figure S2) to a 116-bp minimal notochord CRM (Figure 2A). The minimal 116-bp *Ci-thbs3* notochord CRM contains four Ci-Bra binding sites (numbered T1–4 in Figure 2A), which do not affect notochord activity when individually mutagenized (Figure 2A–2D). However, when three of these sites (T2, T3, and T4 in Figure 2A) are mutated in conjunction, their synergistic activity becomes evident (Figure 2E–2G). These results are quantified in the graph in Figure 2H. In particular, the double mutations of the T2 and T4 sites, which share the same core sequence TCGCAC, and of the T2 and T3 sites (Figure 2E, 2H, and unpublished data) leave only very little notochord activity (red arrowheads in Figure 2E). However, only the triple mutation of sites T2, T3, and T4 completely inactivates the CRM in notochord cells (Figure 2G and 2H). In general, we observed that mutations attacking the “CAC” sequence were usually more effective than the mutations that changed the “T” in the TNNCAC core (unpublished data); hence these mutations were used for all Ci-Bra binding sites analyzed.

### Notochord CRMs Controlled by Ci-Bra through Two Cooperative Binding Sites

**Figure 3:**
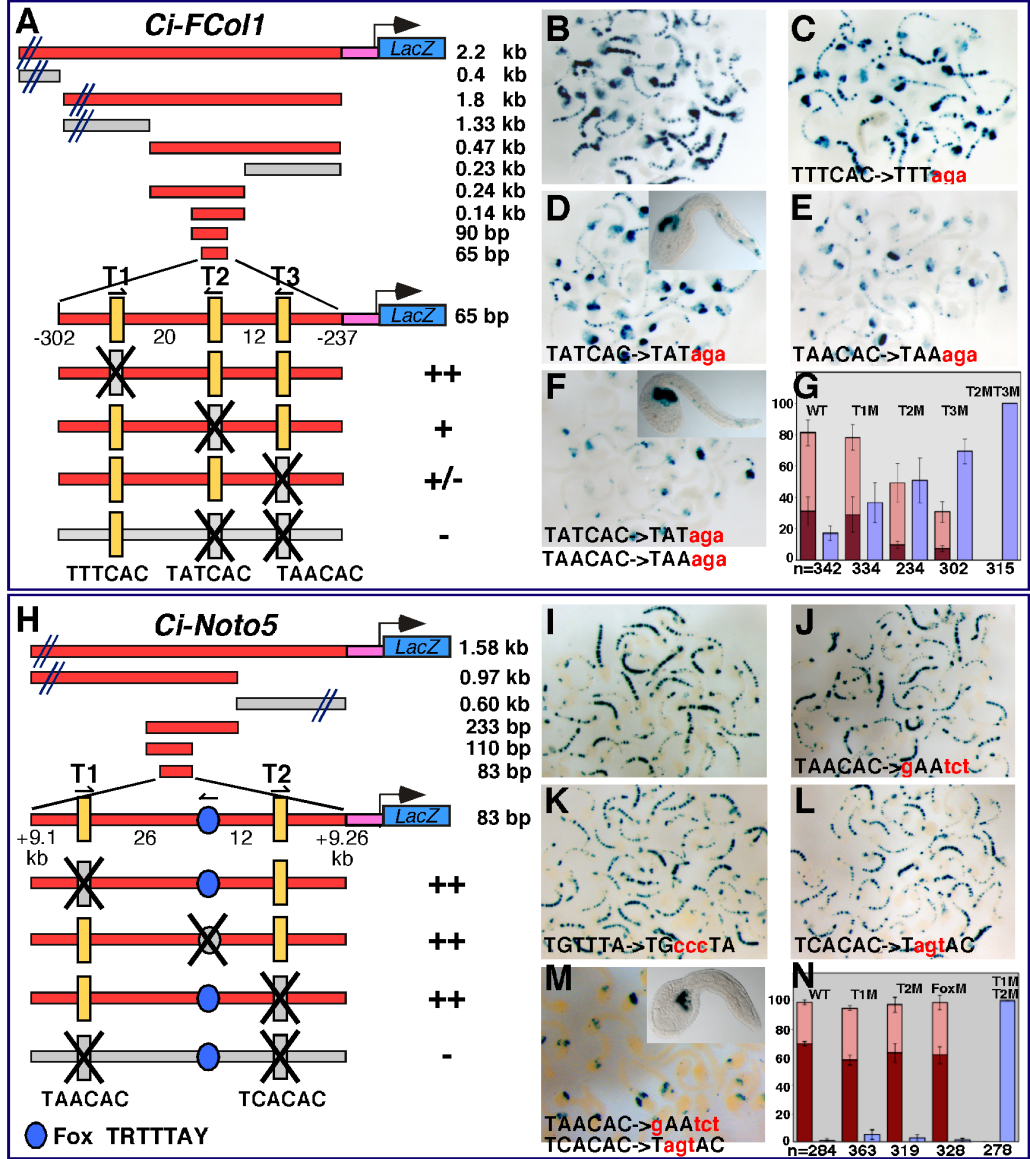
Notochord CRMs controlled by Ci-Bra through two cooperative binding sites. Identification of the minimal sequences necessary for notochord activity of the *Ci-FCol1* and *Ci-Noto5* notochord CRMs, through sequence-unbiased truncation analyses and site-directed mutations. All schematics are as in Figure 2. (A) Sequence-unbiased truncation and site-directed mutation analyses of the *Ci-FCol1* notochord CRM. (B–F) Low-magnification group microphotographs of embryos electroporated in parallel with either the wild-type 65-bp *Ci-FCol1* minimal notochord CRM (B) or with mutated versions of this construct carrying individual mutations in each Ci-Bra binding site, as shown at the bottom of each panel (C–E) or a double mutation in the two proximal Ci-Bra binding sites (F). (G) Quantification of the activity of the constructs shown in (B–F) in different tissues, as in Figure 2. (H) Sequence-unbiased truncation and site-directed mutation analyses of the *Ci-Noto5* notochord CRM. (I–M) Low-magnification group microphotographs of embryos electroporated in parallel with either the wild-type 83-bp *Ci-Noto5* minimal notochord CRM (I) or with mutated versions of this construct carrying the individual mutations in each Ci-Bra binding site shown at the bottom of each panel (J–L) or a double mutation in the two proximal Ci-Bra binding sites (M). (N) Quantification of the activity of the constructs shown in (I–M) in different tissues, plotted as described in Figure 2. Insets show higher magnifications of representative stained embryos.

Another subset of notochord CRMs were also found to require multiple Ci-Bra binding sites for their activity (Figure 3). These notochord CRMs are associated with the genomic loci of *Ci-FCol1* and *Ci-Noto5* and contain Ci-Bra binding sites of various core sequences. The *Ci-FCol1* notochord CRM was originally identified as part of a larger *cis*-regulatory region, spanning 2.2 kb (Figure S2), which also harbors muscle and endoderm CRMs [39]. Truncation analyses allowed the identification of a 65-bp minimal CRM, which contains three Ci-Bra binding sites that were individually mutagenized to assess their respective roles (Figure 3A–3F). Mutation of the distal-most site, with a TTTCAC core, had little or no effect on *cis*-regulatory activity (Figure 3C and 3G), but when similar mutations were introduced in the centrally located Ci-Bra binding site, with a TATCAC sequence, a reduction of notochord activity was detected (Figure 3D and 3G). A stronger effect on notochord staining was seen when the proximal TAACAC site was mutated (Figure 3E and 3G). Finally, the combined mutation of the central and proximal Ci-Bra binding sites was able to completely abolish notochord activity (Figure 3F).

The *Ci-Noto5* notochord CRM, which appeared to be located in the 5′-flanking region of this gene according to outdated gene models, is instead contained in an intron of gene model KH.L153.32 (Figure S2) [40]. The minimal 83-bp *Ci-Noto5* notochord CRM contains two Ci-Bra binding sites at its extremities and a centrally located putative Fox binding site (Figure 3H) with a TRTTTAY core. This sequence was examined because it is shared with the functional Ci-FoxA-a site that is required to activate the *Ci-tune* notochord CRM synergistically with Ci-Bra [41]. Interestingly, none of the individual mutations of either Ci-Bra or Ci-Fox binding sites had any detectable effect on the *Ci-Noto5* CRM notochord activity (Figure 3I–3L); however, the combined mutation of both Ci-Bra binding sites completely abolished staining in notochord cells, leaving the activity in mesenchyme cells intact (Figure 3M). These observations were confirmed by quantitative measurements of a statistically representative number of embryos (Figure 3N).

### Ci-Bra Controls a Subset of Notochord CRMs through Single Binding Sites

**Figure 4:**
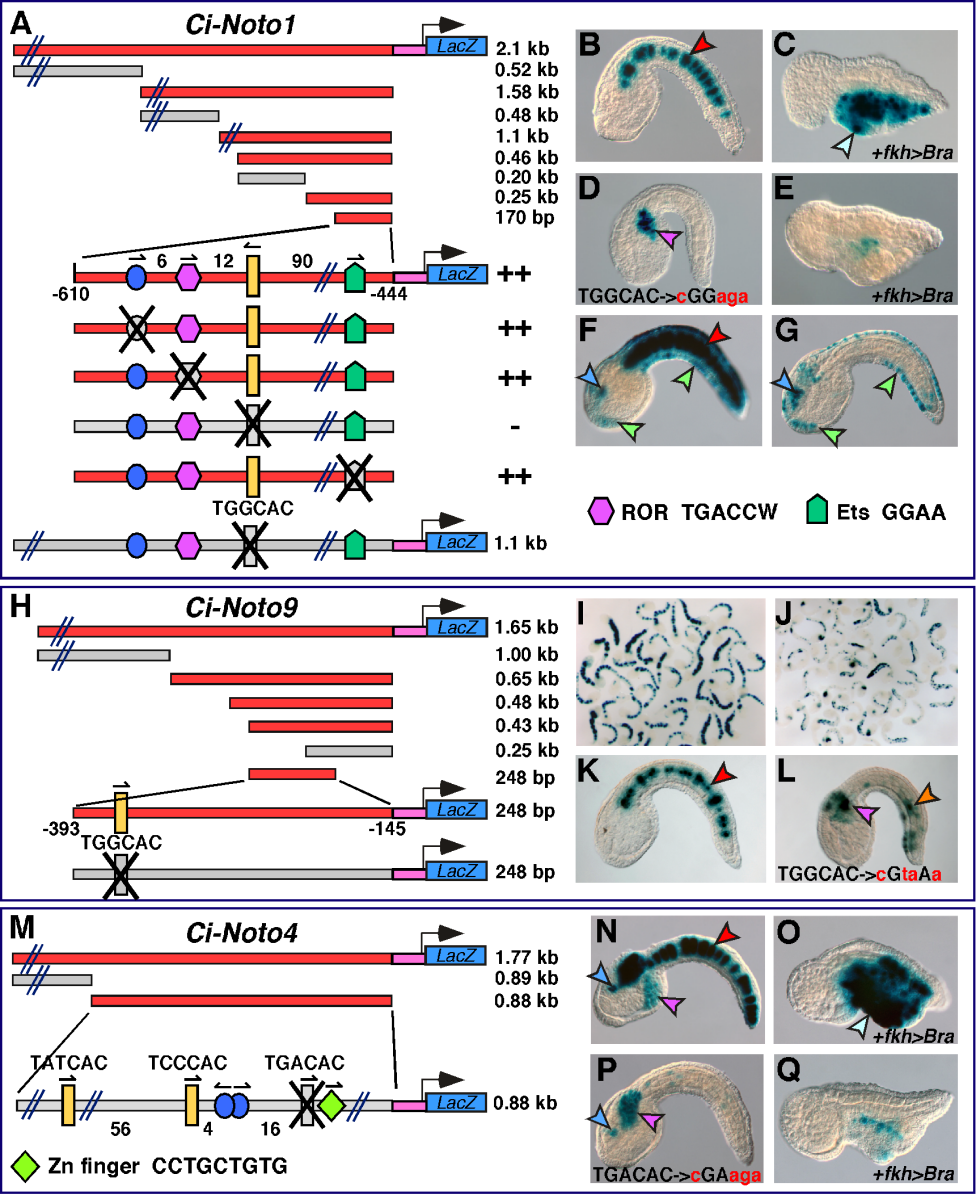
Notochord CRMs controlled by Ci-Bra through single binding sites. (A,H,M) Identification of the minimal sequences necessary for notochord activity of the *Ci-Noto1*, *Ci-Noto9*, and *Ci-Noto4* notochord CRMs, through sequence-unbiased truncation analyses and site-directed mutations. All schematics are as in Figure 2. In the 1.1-kb *Ci-Noto1* construct, only the binding sites that were analyzed are depicted. (B) Control late tailbud embryo carrying the 170-bp minimal *Ci-Noto1* notochord CRM. (C) Embryo from the same clutch as (B), co-electroporated with the 170-bp construct and the *Ci-FoxA-a>Bra* construct; misplaced notochord, endoderm, and CNS cells are indicated by an aqua arrowhead. (D) Control late tailbud embryo carrying the 170-bp minimal *Ci-Noto1* notochord CRM in which the Ci-Bra site shown in (A) has been mutated as shown at the bottom of the panel. (E) Embryo from the same clutch as (D) co-electroporated with the minimal CRM and the *Ci-FoxA-a>Bra* construct. (F) Control embryo carrying a larger 1.1-kb *Ci-Noto1* CRM truncation. (G) Embryo electroporated in parallel with the embryo in (F), carrying the 1.1-kb *Ci-Noto1* CRM truncation with only the Ci-Bra binding site mutated, as shown at the bottom of (A). Note that in the 1.1-kb construct, only the binding sites that were subjected to mutation analysis are depicted. (I,J) Low-magnification group microphotographs of embryos electroporated in parallel with either the 248-bp *Ci-Noto9* minimal notochord CRM (I) or with a version of this construct carrying a mutation in the Ci-Bra binding site (J). (K,L) Individual embryos selected from the experiments shown in (I,J). The mutation of the Ci-Bra binding site is shown at the bottom of (L). (M) Sequence-unbiased truncation analysis of the *Ci-Noto4* notochord CRM. Additional constructs used for the truncation and mutation analysis are shown in Figure S4. In the 0.88-kb construct, only the sites that were analyzed are depicted. (N) Embryo electroporated with the 0.88-kb *Ci-Noto4* CRM fragment shown in (M). (O) Embryo from the same clutch as (N) co-electroporated with the construct in (N) and the *Ci-FoxA-a>Bra* construct. (P) Embryo electroporated with the 0.88-kb *Ci-Noto4* CRM carrying the mutation of a single Ci-Bra binding site, shown at the bottom. (Q) Embryo co-electroporated with the construct in (P) and the *Ci-FoxA-a>Bra* construct. Representative cells of stained tissues are indicated by arrowheads, which are color-coded as follows: red, notochord; blue, CNS; green, epidermis and tail epidermal neurons; purple, mesenchyme; orange, muscle; yellow, endoderm. Green arrowheads are used also to indicate papillary neurons in (F,G).

*Ci-Noto1* becomes detectable in notochord precursors at the late gastrula/early neural plate stage (Figure 1 and [22]). Its notochord expression pattern is recapitulated by a 2.1-kb genomic fragment from its 5′-flanking region (Figure S2). We reduced this fragment through progressive truncations (Figure 4A and 4B) and found that the 170-bp minimal notochord CRM contains putative binding sites for proteins of the Fox, Ets, and ROR transcription factor families. However, differently from the minimal CRMs described thus far, the 170-bp *Ci-Noto1* CRM contains only one putative Ci-Bra binding site. Since previous work had shown that in addition to Ci-FoxA-a and other Fox genes, various members of the Ets and ROR transcription factor families are expressed in the notochord [42], we individually mutagenized these putative binding sites within the 170-bp CRM; however, no reduction of notochord staining was observed (Figure 4A). Instead, the mutation of the single Ci-Bra binding site caused complete loss of notochord activity (Figure 4A and 4D). Considering the fact that *Ci-Noto1* was identified, along with numerous other genes, in a subtractive screen between wild-type and Ci-Bra-overexpressing embryos, we co-electroporated this construct along with *Ci-FoxA-a>Bra*, the construct that was used to induce mis- and over-expression of *Ci-Bra* in endoderm, central nervous system (CNS), and notochord through the *Ci-FoxA-a* promoter region [11], to test whether the 170-bp CRM was responsive to the ectopic expression of Ci-Bra. These experiments demonstrated that the 170-bp CRM is ectopically activated by the misexpression of Ci-Bra (Figure 4C), and that the mutation of the Ci-Bra site abolished this response (Figure 4D and 4E). To further validate these results, the same mutation of the Ci-Bra binding site was introduced into a longer version (1.1-kb) of the CRM, which contains additional Ci-Bra binding sites and directs a more robust staining (Figure 4F). Even within this broader context, the mutation of the single Ci-Bra site was still sufficient to completely obliterate notochord activity, leaving the staining in the CNS and in both papillary and tail neurons unchanged (Figure 4G). This result rules out the possibility that Ci-Bra binding sites found in the longer sequence might be able to compensate for the mutation of the main Ci-Bra binding site, and reinforces the observation that the *Ci-Noto1* CRM relies for activity on a single site.

A Ci-Bra binding site with a core sequence identical to the one identified in the *Ci-Noto1* CRM (TGGCAC) was also found to be necessary for the notochord activity of the CRM associated with *Ci-Noto9* (Figures 4H–4L and S2). *Ci-Noto9* encodes an ortholog of a transcriptional regulator, FUSE-binding protein [43] and is first detected in notochord cells at the neural plate stage (Figures 1 and S1), slightly later than *Ci-Noto1*. Mutation of the Ci-Bra site inactivated the *Ci-Noto9* CRM in notochord cells, leaving intact its ability to sporadically stain mesenchyme and a few muscle cells (Figure 4I–4L).

In addition to the previous cases, another notochord CRM associated with a middle-onset gene, *Ci-Noto4* (Figure S2), was found to be dependent upon an individual Ci-Bra binding site, with core sequence TGACAC (Figure 4M and 4N). Mutation of this site similarly obliterated notochord activity in both the minimal 144-bp CRM and in a longer fragment spanning 0.88 kb (Figure 4M). None of the other putative binding sites identified in the minimal 144-bp CRM, which included putative Fox and Zn-finger binding sites, was found to substantially contribute to the notochord activity upon individual mutagenesis (Figure S4). The mutation of the Ci-Bra binding site was sufficient to abolish both notochord staining and the response to ectopically expressed Ci-Bra (Figure 4N–4Q). Interestingly, a Brachyury binding site with an identical core sequence has been previously described in the enhancer region of *Xenopus Bix4*, a target of Xbra [38].

Finally, the analysis of the weak 972-bp *Ci-Noto8* notochord CRM (Figure S2) identified two Ci-Bra binding sites clustered in tandem arrangement within a 25-bp interval at the 3′-end of this CRM, with core sequences TCACAC and TAACAC (Figure S5). Truncation of the TAACAC site resulted in the inactivation of the CRM in the notochord (Figure S5). We conclude that this site is mainly responsible for the notochord activity of this CRM.

### The Consensus Sequence for Functional Ci-Bra Binding Sites Is Evolutionarily Conserved

The experiments described here allowed us to gather a set of minimal CRM sequences and of functional Ci-Bra binding sites, which are shown in Table 1 along with previously published Ci-Bra binding sites. From this comparison, it is evident that among the 16 possible combinations, some core sequences are preferentially represented in the *Ciona* notochord CRMs identified thus far (Table 1), and that some core sequences are more frequently encountered in functional Ci-Bra binding sites (highlighted in bold in Table 1). Of note, 7 out of 16 (∼44%) of the possible core sequences are yet to be found to be required for notochord activity in any CRM.

**Table 1:** Occurrence and functional requirement of different core T-box binding sites found within notochord CRMs.

Sequence	Representation^a^ and Requirement^b^	Binding Evidence	Reference
TAACAC	***CiFCol1*** **, ** ***Ci-Noto5*** **, ** ***Ci-ABCC10*** **, ** ***Ci-ERM*** **, ** ***Ci-Noto8*** **, ** ***Ci-thbs3*** **, ** ***Ci-Tbx2/3*** ** (2x) ** [**69**], *Ci-Noto8* (3x)	ChIP	[69], this study
TCACAC	***Ci-leprecan*** **** [**70**] **, ** ***Ci-ERM*** **, ** ***Ci-lamc1*** **, ** ***Ci-Noto5***, *Ci-Noto8*	EMSA	[70]
TATCAC	***Ci-leprecan*** **** [**70**] **, ** ***CiFCol1*** **, ** ***Ci-trop*** **** [**71**], *Ci-Noto4*	EMSA	[70],[71]
TCGCAC	***Ci-trop*** **** [**71**] **, ** ***Ci-lamc1*** ** (3′–5′), ** ***Ci-thbs3*** ** (2x)**, *Ci-lamc1* (5′–3′)	EMSA	[71]
TGGCAC	***Ci-Noto1*** **, ** ***Ci-Noto9***, *Ci-thbs3*, *Ci-ABCC10*, *Ci-Noto8*	ChIP	This study
TTGCAC	***Ci-tune*** **** [**72**] ** (5′–3′)**, *Ci-tune* (3′–5′), *Ci-Noto8*	EMSA	[72]
TGACAC	***Ci-Noto4***	ChIP	This study
TTACAC	***Ci-leprecan*** **** [**70**], *Ci-Noto8*	EMSA	[70]
TGTCAC	***Ci-Tbx2/3*** **** [**69**], *Ci-ABCC10*	ChIP	[69], this study
TTTCAC	*CiFCol1*, *Ci-ABCC10*		This study
TCTCAC	*Ci-ABCC10*, *Ci-lamc1*, *Ci-trop* [71]		[71], this study
TACCAC	*Ci-Noto8*		This study
TAGCAC	*Ci-Noto8*		This study
TCCCAC	*Ci-Noto4*		This study
TGCCAC	*Ci-ABCC10*		This study
TTCCAC			

a Representation in plain font.

b Requirement in bold font.

EMSA, electrophoretic mobility shift assay.

We have aligned the functional core Ci-Bra TNNCAC sequences and their flanking regions and we have compared them to the published binding sites for Brachyury proteins identified in other organisms. The most informative alignments are shown in Table 2, where we used as a reference the consensus binding site previously identified in *Drosophila* for the Brachyury ortholog Brachyenteron, which was shown to also be bound by mouse Brachyury [44]. The vast majority of the functional Ci-Bra binding sites identified thus far display a considerable homology with the consensus sequence identified in *Drosophila*, with the mismatches occurring almost exclusively in the outermost flanking nucleotides (highlighted in red in Table 2). In particular, the *Ciona* provisional consensus is richer in pyrimidines at both its 5′ and 3′ ends.

**Table 2:** Comparison of functional Ci-Bra binding sites with the consensus binding site identified for other Brachyury proteins.

Site Sequence	Mechanism	CRM
RWWNTNRCACYT [44]	Cooperative [44]	*Drosophila orthopedia* [44]
tTTTTGGCACCT	Individual	*Ci-Noto1*
GAcATGGCACTT	Individual	*Ci-Noto9*
ATTTTGACACCc	Individual	*Ci-Noto4*
tgATTAACACCT	Individual	*Ci-ABCC10*
GTTTTAACACCa	Individual	*Ci-Noto8*
tcATTTGCACTc	Individual	*Ci-tune* [72]
tcTCTCGCACCc	Cooperative	*Ci-trop* [71]
GTTTTATCACTa	Cooperative	*Ci-trop* [71]
cTACTAACACCa	Cooperative	*CiFCol1*
cAAATAtCACga	Cooperative	*CiFCol1*
AAAGTAACACac	Cooperative	*Ci-Noto5*
AAACTCACACag	Cooperative	*Ci-Noto5*
tAATTAACACgT	Cooperative	*Ci-ERM*
tAcGTCACACTa	Cooperative	*Ci-ERM*
cAATTCACACTT	Cooperative	*Ci-lamc1*
AAcTTCGCACCg	Cooperative	*Ci-lamc1*
tTATTCGCACTg	Cooperative	*Ci-thbs3*
cgAATAACACTT	Cooperative	*Ci-thbs3*
tgTTTCGCACgT	Cooperative	*Ci-thbs3*
cAgTTTACACaa	Cooperative	*Ci-leprecan* [70]
AAAGTAtCACac	Cooperative	*Ci-leprecan* [70]
AgATTAACACTT	Cooperative	*Ci-Tbx2/3* [69]
tTcTTGTCACaT	Cooperative	*Ci-Tbx2/3* [69]
cTTATAACACga	Cooperative	*Ci-Tbx2/3* [69]

Bases that diverge from the consensus are indicated in lower case. The core TNNCAC sequence is underlined.

N, any base; R, A or G; W, A or T; Y, C or T.

We then estimated the distance of the functional Ci-Bra binding sites from the putative transcription start sites of their neighboring genes, in order to identify spatial constraints that might modulate the activity of the functional Ci-Bra binding sites within their genomic context. We referred to the 5′-end of the updated evidence-based KH gene models (http://ghost.zool.kyoto-u.ac.jp/SearchGenomekh.html#CDNA) [40] as the transcription start sites (Table S2). Even taking into account the position bias that characterized the approach used for the identification of the majority of the CRMs, which targeted the 5′-flanking regions, we observed that the Ci-Bra binding sites of single-site CRMs are predominantly located within <600 bp of the respective putative transcription start sites, with the exception of the *Ci-ABCC10* Ci-Bra binding site, which lies at position +2.3 kb. The cooperatively acting binding sites can be found, on average, at higher distances from the transcription start sites, with the *Ci-Noto5* notochord CRM being located >9 kb downstream of the *Ci-Noto5* transcription start site (Table S2). These findings do not reveal evident recurring intervals or other architectural constraints, suggesting that Ci-Bra might be able to activate transcription from its target CRMs regardless of their location within the genomic loci.

### Two Ci-Bra-Downstream Minimal Notochord CRMs Are Devoid of Ci-Bra Binding Sites

**Figure 5:**
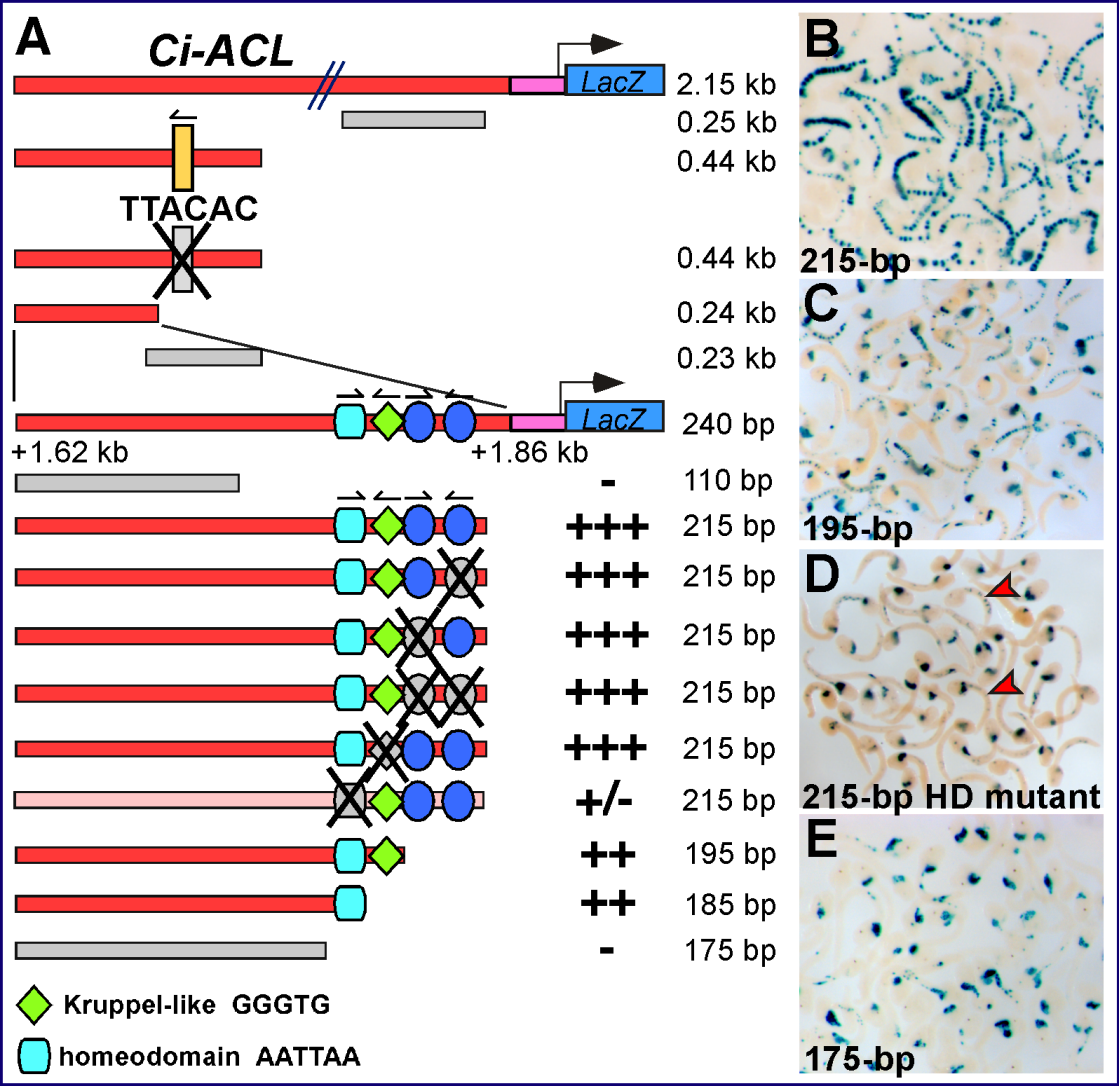
A Ci-Bra-downstream minimal notochord CRM lacking Ci-Bra binding sites. Identification of the minimal sequences required for notochord activity of the *Ci-ACL* notochord CRM. (A) Schematic representation of constructs containing serial truncations and site-directed mutations. (B–E) Low-magnification group microphotographs of embryos electroporated in parallel with either the wild-type 215-bp *Ci-ACL* notochord CRM (B) or 195-bp truncation (C), a mutated version of the 215-bp construct carrying a mutation in the putative Homeodomain (abbreviated as HD) binding site (D), or the 175-bp truncation (E). Red arrowheads in (D) highlight embryos showing residual notochord staining.

The *Ci-ACL* CRM was first identified as a 2.15-kb fragment from the 5′-flanking region of the *Ci-ACL* gene (Figure S2). This region was subsequently reduced through serial truncations to a 215-bp notochord-specific CRM, which differs from the CRMs previously described since it is devoid of apparent Ci-Bra/T-box TNNCAC binding sites (Figure 5A and 5B). Among the recognizable putative binding sites that were found by scanning this sequence were two Fox sites, a Krüppel-like site and a generic homeodomain site, all of which are clustered within 40 bp at the 3′-end of the 215-bp sequence. Mutations of the putative Fox sites, both individually and combined, did not decrease notochord staining, nor did the mutation in the putative Krüppel-like binding site (Figure 5A and unpublished data), although the truncation of the region containing both Fox sites reduced the notochord activity (Figure 5A and 5C). However, a mutation of 4 bp, which changes the AATTAA core binding site for homeodomain proteins to TTTTGC, was sufficient to abolish over 90% of the notochord activity (Figure 5A and 5D). Finally, the truncation of the whole 40-bp 3′-end region of the CRM was able to completely obliterate notochord activity (Figure 5A and 5E). These results suggest that this 215-bp notochord CRM primarily requires a sequence that resembles the binding site for transcription factors of the homeodomain family for its activity; this sequence might function synergistically with sequences contained in the 40-bp 3′-end region, as well as with sequences found at the 5′-end region of the 215-bp CRM, as truncations of this region also weaken the notochord staining, although to a lesser extent (unpublished data).

Similar results were obtained through the dissection of the 2.47-kb notochord CRM identified in the 5′-flanking region of the late-onset gene *Ci-β4GalT* (Figures 1 and S2). The minimal *Ci-β4GalT* CRM is also devoid of Ci-Bra binding sites, although it does not apparently rely upon a homeodomain or any other clearly identifiable binding site (unpublished data). These results bring forth the possibility that Ci-Bra might also be controlling the *Ci-β4GalT* CRM through a transcriptional intermediary, which is likely distinct from the factor that regulates the *Ci-ACL* CRM. This “relay” mechanism is consistent with the late developmental onset of expression of the genes associated with these CRMs, which begin to be expressed at the late neural plate and at the neurula stage, respectively (Figure 1 and [22]).

### The Number of Functional Ci-Bra Binding Sites in a Minimal CRM Correlates with the Developmental Onset of Its Notochord Activity

**Figure 6:**
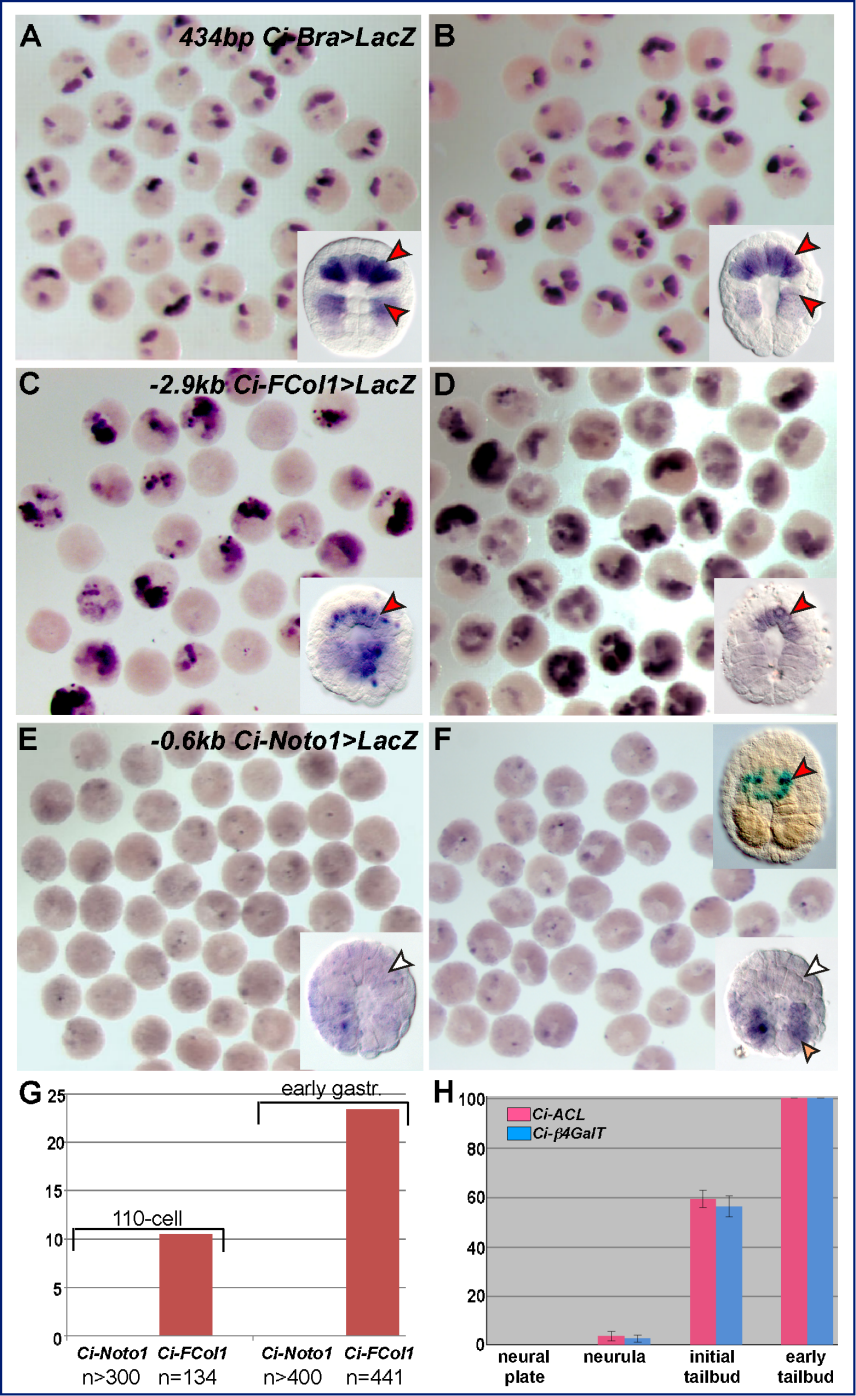
Developmental onsets of activity of notochord CRMs representative of the multiple- and single-site Ci-Bra targets, and of two notochord CRMs lacking Ci-Bra binding sites. Time-course experiments were carried out by WMISH on transgenic embryos (A–F) carrying the notochord CRMs indicated on the top right corner of (A, C, E), fixed at the 110-cell (A,C,E) and early gastrula stage (B,D,F). Insets in the bottom right corners display high-magnification views of representative stained embryos. The inset in the top right corner of (F) shows an embryo carrying the −0.6 kb *Ci-Noto1>LacZ* transgene stained with X-Gal. (G) Graph of a representative time-course experiment, reporting the number of transgenic embryos showing *LacZ* expression in notochord precursors, for the constructs and stages detailed in the panel. *n*, number of scored embryos showing hybridization signal. (H) Graph of time-course experiments for the notochord CRMs associated with the late-onset genes *Ci-ACL* and *Ci-β4GalT*, as determined by X-Gal staining. Results of three representative experiments were averaged. The number of embryos scored for the *Ci-ACL* CRM was: neural plate, *n* = 327; neurula, *n* = 419; initial tailbud, *n* = 389; early tailbud, *n* = 237. The number of embryos scored for the *Ci-β4GalT* CRM was: neural plate, *n* = 342; neurula, *n* = 402; initial tailbud, *n* = 348; early tailbud, *n* = 220.

Once different categories of notochord CRMs were identified, we noticed that single-site minimal CRMs had, on average, the same qualitative “strength” as the multiple-site CRMs, i.e., they were able to direct intense notochord staining in a large number of embryos. Therefore, we investigated whether these different *cis*-regulatory mechanisms rather influenced the developmental onsets of the CRMs. We selected representative CRMs associated with direct Ci-Bra targets of the early-onset and middle-onset groups, and to precisely determine their developmental onsets we re-cloned these CRMs upstream of their endogenous promoters. This strategy was employed to avoid possible interference from the early *Ci-FoxA-a* basal promoter [45] and to recapitulate the natural context of each CRM. We prepared these endogenous promoter constructs for the *Ci-thbs3* and *Ci-FCol1* CRMs (Figure 1), which are associated with early-onset genes, for *Ci-Noto1*, which is linked to a typical middle-onset gene [22], and for *Ci-β4GalT* as a representative late-onset gene. Time-course experiments were carried out for these CRMs, which were all driving the *LacZ* reporter, in parallel with the 434-bp *Ci-Bra>LacZ* CRM [27], which provided a control for temporal onset and notochord-specific staining (Figure 6A and 6B). The results for the *Ci-FCol1* and the *Ci-Noto1* time-courses are shown in Figure 6C–6G. Embryos electroporated with these constructs were allowed to develop until the 110-cell (Figure 6A, 6C, and 6E), early gastrula (Figure 6B, 6D, and 6F), and mid-gastrula stages (unpublished data), then subjected to WMISH using an antisense RNA *LacZ* probe and scored for *LacZ* expression in notochord precursors (Figure 6G). The results revealed a sharp difference between the onset of activity of the *Ci-FCol1* CRM, which is first detected in notochord precursors at the 110-cell stage (Figure 6C and 6G) and increases at the early gastrula stage (Figure 6D and 6G), and the onset of *Ci-Noto1*, whose activity is first detected at the 110-cell stage, weakly and sporadically, in muscle precursors (Figure 6E, 6G, and inset in 6F) and in early gastrulae increases in this territory, while remaining absent from the notochord (Figure 6F and 6G). By late gastrulation, ∼10% of total *Ci-Noto1* transgenic embryos begin displaying 5-bromo-4-chloro-3-indolyl-β-D-galactopyranoside (X-Gal) staining (top right inset in Figure 6F). Early onset similar to that of *Ci-FCol1* was determined through the time-course of *Ci-thbs3*, whose activity was also first detected at the 110-cell stage, as in the case of *Ci-FCol1*, although in a lower number of embryos (2.4% versus 10.4% of total stained embryos; unpublished data). A late onset was also detected for the single-site *Ci-Noto4* CRM, in which case X-Gal staining was detected in 5.2% of the stained neural plate embryos (unpublished data).

Lastly, we carried out a similar time-course experiment using the *Ci-ACL* and *Ci-β4GalT* constructs described in Figures 5 and S2, and we detected X-Gal staining beginning at the neurula stage (Figure 6H). Comparable results were observed for the *Ci-β4GalT* CRM cloned on its endogenous promoter (unpublished data).

Together, these results indicate that notochord CRMs controlled by Ci-Bra through multiple binding sites display the earliest onset of activity, around the 110-cell stage, while the notochord CRMs controlled by Ci-Bra through a single binding site become active around the mid/late-gastrula stage, and the notochord CRMs controlled by Ci-Bra indirectly are activated around neurulation. The comparison between *Ci-FCol1* and *Ci-thbs3* shows that the number of cooperative Ci-Bra binding sites in a CRM does not influence the onset of activity. In conclusion, the time-course experiments indicate that the onsets of activity of the notochord CRMs mirror the onsets of expression of the endogenous genes associated with them (Figures 1 and S1).

### Applicability of the Newly Identified Mechanisms of *cis*-Regulatory Control

**Figure 7:**
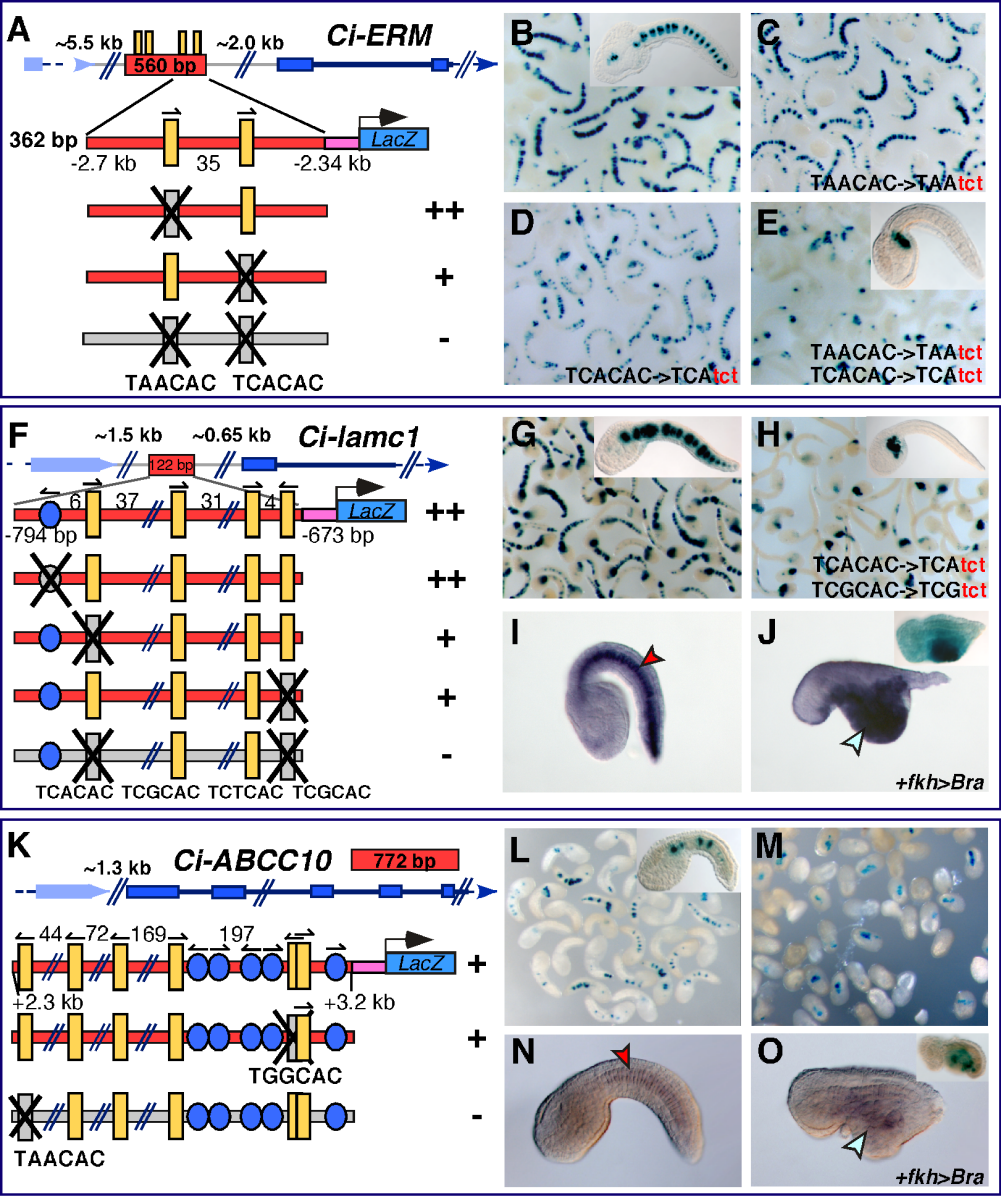
Applicability of the *cis*-regulatory mechanisms identified. (A) A 560-bp notochord CRM (red box) containing Ci-Bra binding sites (yellow vertical bars) was predicted and identified in the 5′-flanking region of *Ci-ERM* and subsequently truncated to a 362-bp sequence containing two Ci-Bra binding sites. (B–E) Late tailbud *Ciona* embryos electroporated with the wild-type 362-bp *Ci-ERM* notochord CRM (B) and its individual (C,D) and double (E) mutant versions. Inset in (E): a representative embryo showing staining only in mesenchyme cells. (F–O) Identification of functional Ci-Bra binding sites in notochord CRMs linked to genes that had not been previously associated with Ci-Bra. (F) Genomic location, structure, and mutation analysis of the notochord CRM identified upstream of *Ci-laminin gamma-1* (*Ci-lamc1*). (G,H) Low-magnification microphotographs of embryos electroporated with the 122-bp *Ci-lamc1* notochord CRM, wild-type (G) or (H) carrying mutations affecting two Ci-Bra binding sites (bottom construct in (F)). Insets in (G,H): individual embryos shown at a higher magnification. (I,J) Late tailbud embryos from the same clutch hybridized *in situ* with the *Ci-lamc1* antisense RNA probe. (I) Control wild-type embryo. (J) Embryo electroporated with the *Ci-FoxA-a>Bra* construct. Inset: late tailbud embryo co-electroporated with the construct in (G) and with *Ci-FoxA-a>Bra*. (K) Genomic location, structure, and mutation analysis of the notochord CRM identified within the *Ci-ABCC10* genomic locus. (L,M) Low-magnification microphotographs of embryos electroporated with the 772-bp wild-type *Ci-ABCC10* CRM cloned on its endogenous promoter. Insets: individual embryos shown at a higher magnification. Inset in (L) displays a representative stained embryo. (N,O) Embryos hybridized *in situ* with the *Ci-ABCC10* antisense RNA probe. (N) Control wild-type embryo. (O) Embryo electroporated with the *Ci-FoxA-a>Bra* construct. Inset: individual embryo co-electroporated with the construct in (L) and with the *Ci-FoxA-a>Bra* construct. The sequences of the additional putative Ci-Bra binding sites found in the 772-bp *Ci-ABCC10* CRM are, from left to right: TCTCAC, TTTCAC, TGTCAC, and TGCCAC. Red arrowheads indicate representative notochord cells, aqua arrowheads indicate the territory where *Ci-Bra* is ectopically expressed.

In an effort to test the general applicability of the *cis*-regulatory mechanisms identified through this study, we employed various combinations of functional Ci-Bra binding sites to rapidly identify genomic regions with the potential to function as notochord CRMs. We first scanned the genomic loci of known Ci-Bra target genes and identified various candidate enhancer regions, among which the most promising was a 560-bp fragment of the 5′-flanking sequence of *Ci-ERM*. This region contained four clustered Ci-Bra binding sites, two of which had identical core sequences and arrangement to those identified in the *Ci-Noto5* CRM (Figure 3H), although with a narrower spacing (45 bp in the case of *Ci-Noto5*, 35 bp in the case of *Ci-ERM*). We cloned and tested this region (Figure 7A) and found that it was sufficient to direct strong notochord expression in *Ciona* embryos. Moreover, when the 560-bp CRM was subdivided into two fragments, we found that only the 362-bp proximal construct, containing the two Ci-Bra binding sites identical to those found in the *Ci-Noto5* CRM, was sufficient to direct strong notochord staining *in vivo* (Figure 7A and 7B). Site-directed mutations of the Ci-Bra binding sites showed that ablation of the distal-most site, TAACAC, did not affect notochord activity (Figure 7C), while the disruption of the proximal Ci-Bra binding site, TCACAC, was able to reduce both the intensity and the frequency of the notochord staining (Figure 7D). However, as in the case of *Ci-Noto5*, the combined mutation of both Ci-Bra binding sites, TAACAC and TCACAC, completely abolished notochord activity, leaving a residual mesenchyme staining and sporadic muscle staining comparable to the vector background staining (Figure 7E and unpublished data). Through time-course experiments, we found that the *Ci-ERM* CRM, once transferred to its endogenous promoter region, began its activity by early/mid-gastrulation (unpublished data).

As a next step, we attempted to extend these predictions to notochord CRMs which we had previously identified, and whose relationship with Ci-Bra was still unclear. We noticed that another minimal 122-bp notochord CRM, which we had identified through a separate set of experiments in the 5′-flanking region of the *Ci-laminin gamma-1* (*Ci-lamc1*) gene, was enriched in Ci-Bra binding sites and also contained a putative Fox binding site (Figure 7F and 7G). After all these sites were separately mutagenized (Figures 7F and S6) and the results were quantified (Figure S6), we found that the simultaneous mutation of two Ci-Bra binding sites, with sequences TCACAC and TCGCAC, respectively, was sufficient to completely abolish notochord activity (Figures 7H and S6), while mutations in the other Ci-Bra binding sites, including another site with an identical TCGCAC core sequence but opposite orientation with respect to the active site, had no visible effect (Figure S6). These data strongly suggested that *Ci-lamc1* could be a notochord gene under the transcriptional control of Ci-Bra. To prove this hypothesis we studied *Ci-lamc1* expression in embryos carrying the *Ci-FoxA-a>Bra* transgene and observed that *Ci-lamc1* is highly responsive to ectopically expressed Ci-Bra (Figure 7I and 7J), as is its notochord CRM (inset in Figure 7J). Of note, after this analysis had been completed, *Ci-lamc1* was also reported as an early Ci-Bra target by another study [13].

Another notochord CRM had been identified in the genomic locus of *Ciona ATP-binding cassette subfamily C member 10* (*Ci-ABCC10*) through a screen of random *Ciona* genomic fragments (unpublished data). Interestingly, this is the first evidence, to our knowledge, of the expression of this transporter protein in the notochord. We had previously narrowed the original 2.146-kb sequence to a 772-bp fragment (Figure 7K, 7L, and unpublished data) via sequence-unbiased truncations, and sequence inspection revealed that this shorter CRM fragment contained six putative Ci-Bra binding sites (listed in Table 1). We focused the point-mutation analyses on the two sites that had been found to be required for the activity of other notochord CRMs. Surprisingly, the mutation of the TGGCAC site, which is necessary for the *Ci-Noto1* and *Ci-Noto9* CRMs (Figure 4), did not affect notochord staining (Figure 7K and unpublished data), while the mutation of the distal TAACAC site was sufficient to completely abolish notochord activity, but left the mesenchyme staining unaffected (Figure 7K, 7L, and unpublished data). On the basis of its dependence upon a single functional Ci-Bra binding site, we predicted this notochord CRM to behave as a middle-onset. To verify this point, we cloned the CRM upstream of its endogenous promoter region and carried out time-course experiments, as previously described. Through WMISH (unpublished data) and X-Gal staining, we determined that the onset of activity of this weak CRM is around the neural plate/early neurula stage, when notochord activity is detected in >50% of the stained embryos (unpublished data); notochord staining increases at the initial tailbud stage (Figure 7M). This is consistent with the timing of *Ci-ABCC10* transcript accumulation in the notochord (Figure S7). To assess the hierarchical relationship of *Ci-ABCC10* with Ci-Bra, we studied *Ci-ABCC10* expression in embryos carrying the *Ci-FoxA-a>Bra* transgene and found that this gene is ectopically expressed in response to the ectopic expression of *Ci-Bra* (Figure 7N and 7O); a similar behavior was exhibited by the *Ci-ABCC10* notochord CRM in *Ci-FoxA-a>Bra* embryos (inset in Figure 7O).

### *In Vivo* Occupancy of the Newly Identified Notochord CRMs by Ci-Bra

**Figure 8:**
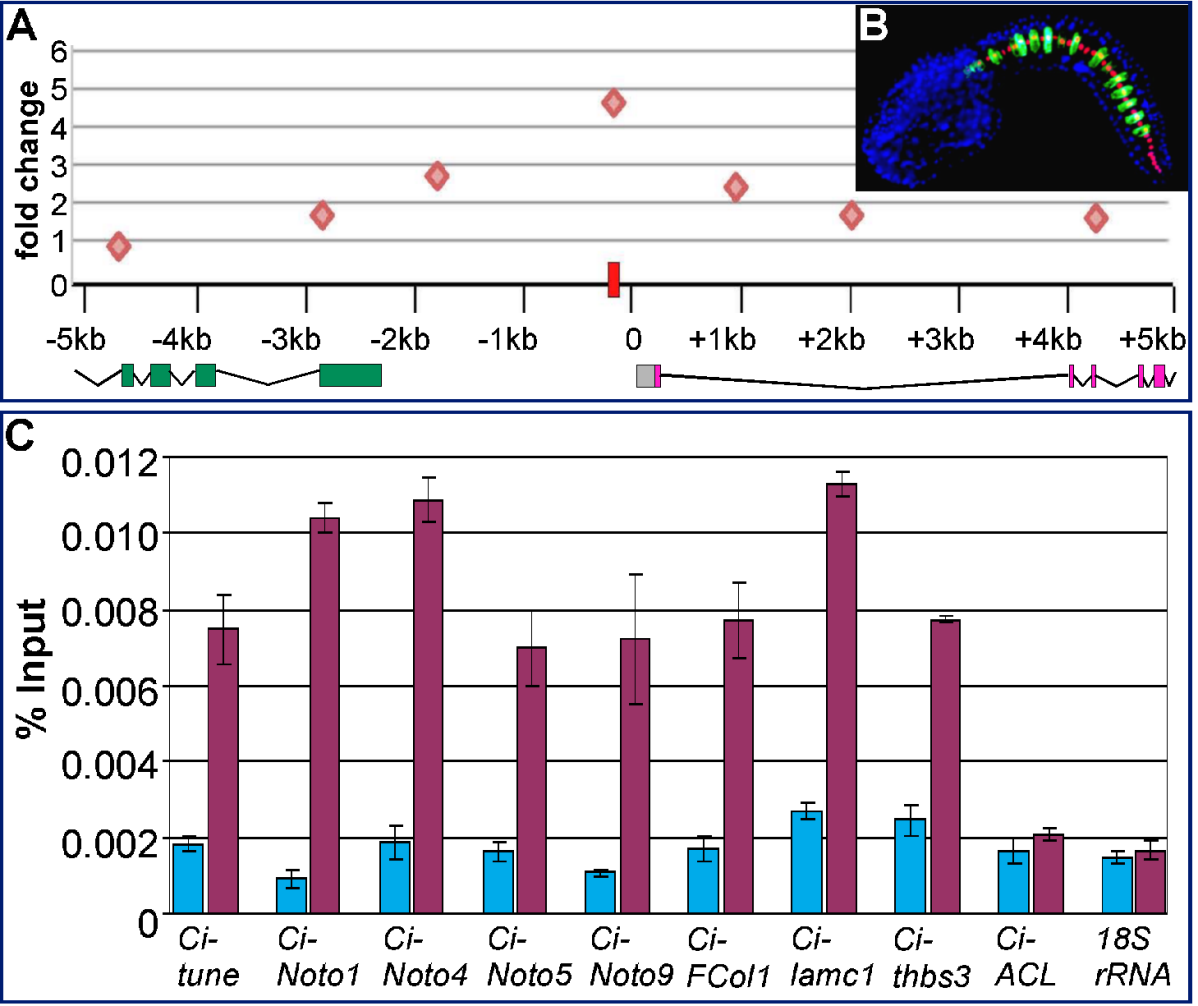
Validation of the *in vivo* occupancy of the Ci-Bra target notochord CRMs through ChIP assays. (A) Graph of ChIP-qPCR experiments carried out using the antibody shown in (B) on various sequence stretches from the *Ci-FCol1* genomic locus and its neighboring gene, KH.C7.121. Orange diamonds indicate peaks of Ci-Bra occupancy, measured as fold change between Ci-Bra-immunoprecipitated and IgG-immunoprecipitated chromatin. A red rectangle shows the location of the *CiFCol1* notochord CRM. Colored boxes below the graph symbolize exons (green, gene KH.C7.121; grey, 5′-UTR; pink, *Ci-FCol1*); the lines connecting them represent introns. (B) Fluorescence microphotograph of a late tailbud *Ciona* embryo carrying the *Ci-Bra>GFP* transgene [27], immunostained with the Ci-Bra-specific antibody [46]. The nuclei of the 40 notochord cells are stained by the antibody in red; GFP expression is detected in a subset of the 40 notochord cells, owing to mosaic incorporation of the transgene. Nuclei in tissues other than the notochord are stained with 4′,6-diamidino-2-phenylindole (DAPI) (blue). (C) Graph showing the results of ChIP-qPCR experiments on the notochord CRMs indicated on the *x*-axis, reporting the enrichment of the immunoprecipitated DNA over the input. All *p*-values were <0.01, with the exception of *Ci-ACL* and *Ci-18S RNA*, which had non-significant *p*-values (negative controls). The primers used are listed in Table S3. Light blue bars, negative controls, i.e. ChIP assays executed with IgG. Magenta, ChIP performed with the Ci-Bra antibody shown in (B).

We had tested in previous studies some of the putative Ci-Bra binding sites identified in notochord CRMs for their ability to be bound *in vitro* by Ci-Bra via electrophoretic mobility shift assays (EMSA) (Table 1); here we assessed the occupancy of these sites *in vivo* through ChIP assays on mid-tailbud stage embryos (Figure 8A) using a polyclonal Ci-Bra antibody (Figure 8B) [46]. To test the specificity of the binding by Ci-Bra, we carried out ChIP over a 10-kb stretch encompassing part of the *Ci-FCol1* locus and its neighboring gene (Figure 8A). The results show that the highest peak of Ci-Bra occupancy indeed corresponds to the *Ci-FCol1* notochord CRM (red rectangle in Figure 8A); these results are comparable to those previously published [13], although not all peaks of Ci-Bra occupancy coincide (unpublished data), likely because our experiments were carried out on embryos at a much later stage (mid-tailbud versus 110-cell). We then proceeded with the ChIP assays of the remaining notochord CRMs, along with adequate controls (Figure 8C and unpublished data). The results demonstrated that in addition to the *Ci-tune* minimal CRM [41], which served as one of our positive controls, the *Ci-Noto1*, *Ci-Noto4*, *Ci-Noto5*, *Ci-Noto9*, *Ci-FCol1*, *Ci-lamc1*, and *Ci-thbs3* CRMs are also bound *in vivo* by Ci-Bra. On the other hand, the minimal *Ci-ACL* notochord CRM, which we have shown to be devoid of Ci-Bra binding sites, was not specifically recognized, similar to the negative control used for these experiments, *18S rRNA* gene. These results confirm the findings obtained through the analysis of individual CRMs. No direct relationship was observed between the number of functional Ci-Bra sites and the enrichment of the immunoprecipitated DNA over the input; this might be due to the size of the immunoprecipitated fragments (∼200–800 bp on average), which likely contain additional sequences that are bound by Ci-Bra but are not required for the activity of the minimal CRMs.

### Phylogenetic Footprinting of Minimal Notochord CRMs Reveals High Variability in the Interspecific Conservation of Functional Ci-Bra Binding Sites

We analyzed the VISTA phylogenetic footprints (http://pipeline.lbl.gov/cgi-bin/gateway2) obtained by comparing the sequences of the notochord CRMs identified in this study between *C. intestinalis* and *C. savignyi* to assess the extent of their evolutionary conservation. We found that the most highly conserved notochord CRMs are *Ci-Noto9* (single-site) and *Ci-FCol1* (multiple-site) (Figure S8). In the case of *Ci-Noto9*, the conservation of the functional Ci-Bra site and its flanking sequences between the two species is complete. In the case of the other single-site CRMs, we found that in the *C. savignyi Noto1* genomic region corresponding to the *C. intestinalis* CRM there was a single change in the TNNCAC core sequence, which did not disrupt the putative Brachyury binding site (TGGCAC to TGCCAC). Sequence comparisons for *Ci-Noto4* showed a disruption of the functional Ci-Bra site(s) sequence found in the corresponding location (TGACAC to TCACGC) but a surprising conservation of the dispensable TCCCAC site, which suggests that this sequence might be of some relevance in *C. savignyi*. Also in the case of *Ci-Noto8*, the main Ci-Bra binding site was not conserved (TAACAC to TAACAT), although some of the other putative binding sites found in the CRM were shared between the two *Ciona* species (unpublished data). The *Ci-thbs3* CRM displays a complete conservation of one of its three cooperative sites, TAACAC, but disruption of the other two sites, TCGCAC and TGGCAC. As for the multiple CRMs, in the case of *Ci-FCol1*, the dispensable TTTCAC site (Figure 3A) is not conserved, while of the two cooperative sites, one is entirely conserved, but the other has a single nucleotide substitution in *C. savignyi* (TATCAC to TCTCAC), which does not disrupt the TNNCAC sequence and might be therefore a functional binding site in this species. These observations correlate with the results of the point mutation analysis (Figure 3B–3E), which shows that the mutation of the most conserved site, TAACAC, is more effective than the mutation of the less conserved one. The *Ci-ERM* and *Ci-ACL* CRMs showed scattered interspecific conservation, although not in the regions directly corresponding to the functional Ci-Bra binding sites or the putative homeodomain binding site, respectively (unpublished data). Finally, for *Ci-Noto5* we did not find any informative sequence alignment in the regions of the *C. savignyi* locus corresponding to the *C. intestinalis* CRM, and the minimal *Ci-ABCC10*, *Ci-β4GalT*, and *Ci-lamc1* CRMs were poorly conserved overall (unpublished data).

### Changes in the Number of Functional Ci-Bra Binding Sites Are Sufficient to Convert an Early-Onset into a Middle-Onset Notochord CRM

**Figure 9:**
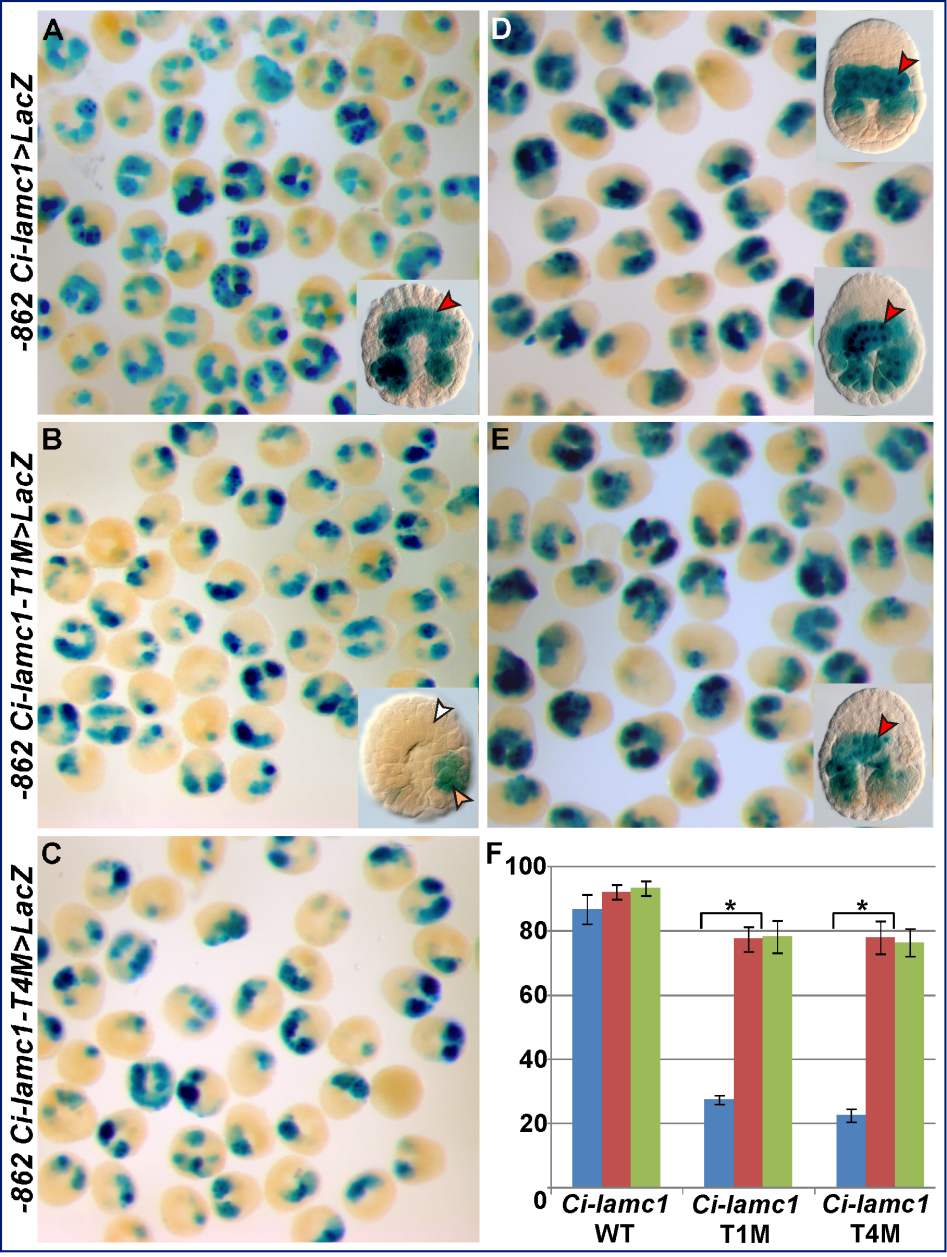
Effects of the removal of functional Ci-Bra binding sites on the developmental onset of a multiple-site notochord CRM. (A–E) Low-magnification microphotographs of *Ciona* embryos carrying either the wild-type *Ci-lamc1>LacZ* notochord CRM transgene (A,D) or a mutated version of this CRM lacking its distal-most Ci-Bra binding site (*Ci-lamc1-T1M>LacZ*; see Figure 7F) or its proximal Ci-Bra binding site (*Ci-lamc1-T4M>LacZ*; see Figure 7F), fixed at the early gastrula (A–C) and mid-gastrula (D,E) stages, and stained with X-Gal. Insets in the low right corners show high-magnifications view of representative stained embryos. The inset in the top right corner of (D) shows an embryos carrying the *434-bp Ci-Bra>LacZ* transgene, which was used to label the notochord lineage at this stage for comparison. Red and white arrowheads: notochord staining, or lack thereof, respectively; orange: muscle staining. (F) Graph showing the percentage of embryos showing notochord staining as a fraction of the total number of stained embryos scored. Blue bars, embryos at the early gastrula stage; brown, embryos at mid-gastrula; green, embryos at the late tailbud stage (see Figure S6). The number of embryos scored for each construct was: WT, early gastrula, *n* = 646; mid-gastrula, *n* = 672; mid-tailbud, *n* = 502. T1M, early gastrula, *n* = 705; mid-gastrula, *n* = 994; mid-tailbud, *n* = 449. T4M, early gastrula, *n* = 756; mid-gastrula, *n* = 700; mid-tailbud, *n* = 431. Statistically significant *p*-values are indicated by asterisks.

Our findings on multiple and single-site CRMs directly controlled by Ci-Bra imply that the conversion of a multiple-site CRM to a single-site should suffice to delay the developmental onset of its activity, thus turning it from an early-onset to a middle-onset CRM. To prove this point, we performed time-course experiments using the *Ci-lamc1* notochord CRM (Figures 7F–7J and S6), after re-cloning it upstream of its endogenous promoter. On the basis of its reliance upon two functional Ci-Bra sites (Figure 7F), we predicted this CRM to display an early onset of activity. In support of this hypothesis, >80% of mid-gastrula embryos electroporated with this construct showed X-Gal staining in notochord precursors (Figure 9A and 9F), indicating that by the early gastrula stage transcription of *LacZ* directed by the *Ci-lamc1* CRM/promoter has already begun. This latter inference was confirmed by *LacZ* WMISH experiments (unpublished data). These experiments indicate that the developmental onset of the *Ci-lamc1* CRM/promoter is comparable to that observed in the case of *Ci-FCol1* (Figure 6C, 6D, and 6G). At the neural plate stage, the percentage of *Ci-lamc1* transgenic embryos with notochord staining was slightly higher (Figure 9D and 9F). However, when we tested constructs carrying mutations in either one of the Ci-Bra functional sites (*Ci-lamc1-T1M* and *Ci-lamc1-T4M*, Figure 9B and 9C) we found that the number of embryos showing notochord staining at the mid-gastrula stage had dropped below 30% compared to the wild-type CRM (Figure 9F). By the neural plate stage, the number of embryos showing notochord staining had significantly increased in both mutants (Figure 9E, 9F, and unpublished data), and had practically reached the levels observed in mid-tailbud stage embryos for these mutants (Figure 9F).

**Figure 10:**
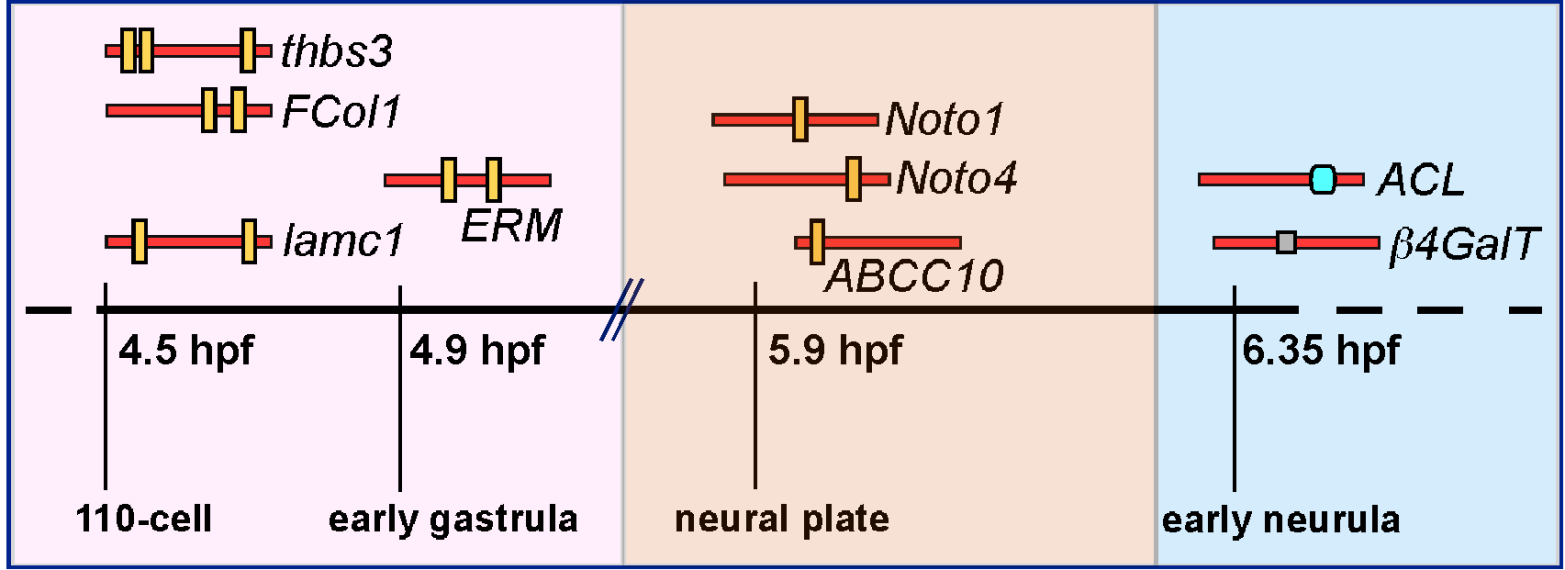
Different structural features of notochord CRMs associated with early, middle, and late-onset Ci-Bra targets. Summary of the developmental onsets of different Ci-Bra targets plotted against the developmental stages indicated at the bottom. Red bars symbolize minimal notochord CRMs; yellow vertical bars indicate functional Ci-Bra binding sites; a blue and a grey square represent binding sites for transcription factors other than Ci-Bra. CRM structures and sizes are approximate, for simplicity. (Left) The notochord CRMs associated with the early-onset genes *Ci-thbs3*, *Ci-FCol1*, *Ci-ERM*, and *Ci-lamc1* are controlled by Ci-Bra through multiple functional binding sites. (Center) The notochord CRMs associated with the middle-onset genes *Ci-Noto1*, *Ci-Noto8*, *Ci-Noto4*, *Ci-Noto9*, and *Ci-ABCC10* are controlled by Ci-Bra through one main binding site. (Right) The minimal notochord CRMs associated with the late-onset genes *Ci-ACL* and *Ci-β4GalT* are controlled indirectly by Ci-Bra via a relay mechanism.

Figure 10 summarizes our findings and proposes a correlation between the number of functional Ci-Bra binding sites and the developmental onsets of the notochord CRMs and the genes linked to them. Notochord CRMs controlled directly by Ci-Bra through multiple functional sites begin their activity between the 110-cell stage and early gastrula, while direct target CRMs controlled by Ci-Bra through a single site become active between late gastrula and neural plate. Finally, notochord CRMs controlled indirectly by Ci-Bra through transcriptional intermediaries begin their activity at neurulation.

## Discussion

Fast-developing embryos usually rely upon maternally stored transcripts and proteins and on shallow gene regulatory networks for the rapid completion of their early morphogenetic processes. For these reasons, it is generally assumed that in ascidians most transcription factors control their target genes directly [47]. Nevertheless, since the initial identification of the first Ci-Bra target genes, their staggered pattern of transcriptional activation throughout development suggested that Ci-Bra controls these genes through different *cis*-regulatory mechanisms. This study presents a first validation of this hypothesis and shows the remarkable variety of alternative mechanisms employed by Brachyury to control its direct and indirect effectors in a simple chordate. In addition, it outlines a connection between different modes of transcriptional regulation and the temporal onset of the genes that they control.

### Variability in the Developmental Onsets of Expression of Ci-Bra Targets in the Notochord

Among the validated Ci-Bra targets expressed in the notochord, the earliest is the planar cell polarity gene *Ci-pk*, which is required for the establishment of notochord cell polarity [48] and for intercalation [23]. *Ci-ERM*, which is first detected in the notochord a few cell divisions after *Ci-pk*, has been shown to be required for notochord elongation [23] and lumen formation [49]. *Ci-Noto4* is first detected in notochord cells at the neural plate stage, and it is also required for midline intercalation [50],[51], as is the late-onset gene *Ci-ACL*, which plays a role also in medio-lateral polarization of notochord cells [23]. *Ci-lamc1* encodes a putative ortholog of human Laminin gamma 1, which is also found in the notochord remnants of the intervertebral discs in human embryos [52]. *Ci-ABCC10* had not been previously detected in notochord cells, and encodes an anionic pump that might be involved in lumen formation, the terminal step of notochord differentiation [30]. Interestingly, this gene is not in the list of putative Ci-Bra targets identified via genome-wide studies of chromatin occupancy in early embryos [13], most likely because its expression begins around neurulation.

These findings underscore the breadth of Brachyury functions, which encompass all stages of notochord formation, and explain the deleterious effects of its inactivation on the development of this structure in widely different chordates, from ascidians [3] to mice [53]. We sought to shed light on the molecular mechanisms that enable Ci-Bra to sequentially deploy its target genes through the systematic characterization of their notochord CRMs.

### The Notochord CRMs Directly Controlled by Ci-Bra Fall into Different Classes

The dissection of notochord CRMs associated with representative Ci-Bra targets allowed their categorization on the basis of the mechanisms employed by Ci-Bra to control their minimal sequences. We found that four CRMs, *Ci-Noto1*, *Ci-Noto9*, *Ci-Noto4*, and *Ci-ABCC10*, are controlled by Ci-Bra through individual binding sites, which are necessary to elicit notochord activity and to mediate the response to ectopically expressed Ci-Bra. These Ci-Bra binding sites have either the core sequence TGGCAC (*Ci-Noto1* and *Ci-Noto9*) or TGACAC (*Ci-Noto4*), while the Ci-Bra binding site in the *Ci-ABCC10* CRM has a TAACAC core. Of note, our results show that in the case of these CRMs, additional Ci-Bra binding sites that might be present in the vicinity of the single functional site are unable to compensate for its loss.

In addition to single-site Ci-Bra target CRMs, we have also identified direct targets that are controlled through two cooperative Ci-Bra binding sites, *Ci-FCol1*, *Ci-Noto5*, *Ci-ERM*, and *Ci-lamc1*. This class also includes the previously characterized *Ci-leprecan* notochord CRM ([26] and unpublished data), the *Ci-trop* notochord CRM, which mainly relies upon a TCGCAC site [21] but also on an adjacent TATCAC site, which alone is not sufficient for activity (our unpublished results). Finally, the *Ci-thbs3* CRM is controlled by Ci-Bra through three binding sites and the *Ci-pk* CRM likely relies upon multiple Ci-Bra binding sites and additional sequences.

Through the analysis of the *Ci-tune* notochord CRM, we had previously identified another class of direct Ci-Bra target CRMs, which are controlled synergistically by Ci-Bra and Ci-FoxA-a [41]. Notably, in the present study, we found that most notochord CRMs contained putative Ci-FoxA-a binding sites in addition to the Ci-Bra binding sites. However, site-directed mutation analyses of the Ci-FoxA-a binding sites that were related to the sites found in the *Ci-tune* CRM (TRTTTAY core) did not reveal an evident role in notochord activity. Nevertheless, it is conceivable that these sites might be used *in vivo* by Fox proteins, which are known to possess a pioneer chromatin-opening activity [54], to increase the accessibility of the CRMs within their native genomic context, or that some divergent Fox binding sites might be contributing to notochord activity.

### Identification of Functional Ci-Bra Binding Sites Required for Gene Expression in Notochord Cells

Recent genome-wide ChIP-chip studies have elucidated the mesodermal gene regulatory network presided over by one of the zebrafish Brachyury orthologs, No tail, leading to the identification of an *in vivo* binding site for this transcription factor, TCACACCT [12],[55], which matches the half-site previously identified for mouse Brachyury [56] and the Xbra binding site identified 936 bp upstream of the promoter region of *Xenopus eFGF* [37]. The present study revealed a considerable heterogeneity in the functional sequences found in direct Ci-Bra targets, as well as the lack of considerable homology in the sequences flanking Ci-Bra binding sites with identical cores. Nevertheless, most of the Ci-Bra functional sequences identified by this and our previous studies conform, albeit to a different extent, to the 12-bp consensus identified for Brachyenteron and vertebrate Brachyury proteins by [44], with the Ci-Bra binding sites seeming prone to higher variability in the nucleotides more distant from the central 6-bp core sequence.

Similarly to its orthologs characterized in other model systems, Ci-Bra is able to bind palindromic sites, possibly in the form of a dimer [21], with the dimerization likely being mediated by the evolutionarily conserved PDSPNF amino acid motif within its T-domain [44]. However, like previously characterized Ci-Bra target CRMs, the CRMs reported here predominantly rely upon half-sites. Only the *Ci-Noto1* functional Ci-Bra binding site, one of the cooperative *Ci-Noto5* sites and one functional Ci-Bra site found in the proximal region of the *Ci-lamc1* CRM display an incomplete palindromic arrangement, which nevertheless seems dispensable for their function, as indicated by the results of individual mutations.

### Structural Features of Predicted Notochord CRMs: Context-Dependent Activity and Architectural Flexibility of Functional Ci-Bra Binding Sites

We tested the general validity of the molecular mechanisms identified through these studies by using the sequences of the functional Ci-Bra binding sites to scan either the loci of other *bona fide* Ci-Bra target genes, or other notochord CRMs identified through cloning of random genomic sequences. Among the interesting clusters of putative Ci-Bra sites that we identified within these sequences, a region found upstream of *Ci-ERM* showed a striking similarity with the *Ci-Noto5* notochord CRM and was therefore cloned and tested *in vivo*, and resulted capable of directing strong notochord staining. Mutation analyses showed that also this predicted CRM relies upon two Ci-Bra binding sites, whose core sequences and arrangement are identical to those of the functional *Ci-Noto5* sites. Of note, the expression patterns of *Ci-Noto5* and *Ci-ERM* in the notochord are remarkably similar (Figure S1). The spacing between the Ci-Bra binding sites varies by 10 bp (35 bp for *Ci-ERM* and 45 bp for *Ci-Noto5*), a full helical turn [57], which seemed suggestive of some flexibility in their spacing. However, when we decreased the distance between the Ci-Bra binding sites in the *Ci-Noto5* CRM to match the spacing found in the *Ci-ERM* CRM, we observed a loss of notochord activity (unpublished data).

When we attempted to predict the functional sites of notochord CRMs identified either through random testing of genomic sequences (*Ci-ABCC10*) or by position-biased cloning (*Ci-lamc1*), again we observed a striking context-dependent difference in the functional relevance of binding sites with identical cores. In fact, in the case of the *Ci-ABCC10* notochord CRM the site with the TGGCAC core, which alone is necessary for the activity of both *Ci-Noto1* and *Ci-Noto9* CRMs, turned out to be fully dispensable, while the mutation of a TAACAC core site was sufficient to abolish notochord activity. Similarly, within the *Ci-lamc1* CRM, the mutations of two identical TCGCAC core sites had very different results, with the distally located site being completely dispensable and the proximal site being highly relevant for notochord activity. Despite the context dependence and the variability in the sequence and relevance of the core Ci-Bra binding sites, a common consensus sequence for functional Ci-Bra binding sites is beginning to emerge from these results; this sequence displays evolutionary conservation with the 12-bp sequence bound *in vitro* by both chordate and protostome Brachyury orthologs [44].

A considerable level of structural flexibility was also observed when the distances of functional Ci-Bra binding sites from the respective transcription start sites were compared. Moreover, our preliminary analyses indicate a remarkably wide degree of variability in the evolutionary conservation of the minimal CRM sequences between the two *Ciona* species. However, it is still possible that some of the Ci-Bra binding sites that are not evidently conserved might be functionally replaced by related sites found in the *C. savignyi* sequences, as we previously hypothesized in the case of the functionally conserved *Ci-tune* CRM [41].

### Identification of Minimal Notochord CRMs Lacking Ci-Bra Binding Sites

In addition to identifying notochord CRMs directly targeted by Ci-Bra, this study has also led to the discovery of two notochord CRMs that are physically associated with *bona fide* Ci-Bra target genes but are devoid of Ci-Bra binding sites in their minimal sequences. These CRMs are associated with two late-onset Ci-Bra targets, *Ci-ACL*, which is activated in notochord at low levels beginning around the late neural plate stage ([22] and our unpublished results) and *Ci-β4GalT*, which is detected in notochord cells starting at the neurula stage. Therefore it seems conceivable that these minimal CRMs might be controlled by Ci-Bra indirectly through transcriptional intermediaries. We have previously shown that transcription factors of different families are expressed in the *Ciona* notochord following the onset of Ci-Bra expression, and that the expression of several of these genes is controlled by Ci-Bra [25],[31]; these reports, along with the results of genome-wide screens [13],[42],[58] provide a number of candidate activators for these CRMs.

The lack of binding by the anti-Ci-Bra antibody that we observed through the ChIP assays suggests that in addition to lacking canonical Ci-Bra binding sites, the *Ci-ACL* notochord CRM is not likely to contain low-affinity, non-canonical Ci-Bra binding sites such as the “type B” sites described in *Drosophila*, which are occupied by Brachyenteron but are unable per se to activate transcription [44]. Rather, this might be the case for the *Ci-β4GalT* CRM, which yielded a relatively noisy ChIP signal (unpublished data). These latter results are consistent with reports in *Drosophila* that show that transcription factors can bind up to thousands of genomic regions which often are not involved in transcriptional events and might be non-functional [59],[60].

### Distinct *cis*-Regulatory Mechanisms Correlate with the Sequential Developmental Onsets of Notochord Gene Expression

The results of this study indicate that the notochord CRMs controlled by Ci-Bra through multiple sites are frequently linked to genes that begin to be expressed early during notochord development, around the early/mid-gastrula stage, and are usually expressed at high levels in these cells. Instead, the notochord CRMs controlled by Ci-Bra through single sites are usually associated with genes that are expressed in the notochord at low levels starting around the late gastrula/neural plate stage. This hypothesis is confirmed by the observation that the removal of one of the functional Ci-Bra binding sites from the multiple-site *Ci-lamc1* CRM results in a delay in the developmental onset of its notochord activity. One possible scenario arising from these observations suggests that by the 110-cell stage, at a time when a substantial accumulation of Ci-Bra in the nuclei is detected (our unpublished results), the synergistic binding of Ci-Bra molecules to the multiple-site CRMs might trigger faster changes in the chromatin state and in the rate of transcriptional activation, as compared to the binding to an individual main site. Hence, transcripts for genes controlled through multiple-site CRMs are detected earlier and in larger amounts than those linked to single-site CRMs. These results might enable predictions of the expression patterns of Brachyury target genes in *Ciona* and possibly in other chordates. To our knowledge, this represents the first report of a correlation between the number of functional Brachyury binding sites found in minimal CRMs and the onset of transcription of their target genes in a chordate. In non-chordate model systems, it is noteworthy that another transcriptional activator, the ortholog of FoxA2/HNF3beta, is able to modulate the onset of gene expression in the pharynx of the nematode *Caenorhabditis elegans* through binding sites with different affinity [61]. High-affinity binding sites are associated with early expression and can be activated by low levels of FoxA2/HNF3beta during early organogenesis [61]. This mechanism therefore resembles the spatial read-outs observed in the *Drosophila* embryo in response to the anterior-posterior and dorsal-ventral gradients of the morphogens Bicoid [62] and Dorsal [63], respectively. Our findings on the context-dependent behavior of Ci-Bra binding sites with identical TNNCAC core sequences suggest that additional sequences, rather than the core sequences per se, might be modulating the binding affinity of Ci-Bra for its sites. Preliminary evidence suggests that a subset of the TNNCAC Ci-Bra binding sites found in single-site notochord CRMs share short flanking sequences, which could be responsible for their indispensable function (DSJ-E and ADG, unpublished observations).

We conclude that even though Ci-Bra controls several of its target genes via direct binding, this shallow branch of its gene regulatory cascade is modulated at the level of individual CRMs by the differential mechanisms identified through this study, to ultimately result in a gradated developmental response.

## Methods

### Animal Husbandry, Electroporation, and Scoring Of Stained Embryos

Adult *C. intestinalis* were purchased from Marine Research and Educational Products (M-REP) and kept in an aquarium in recirculating artificial sea water at ∼18°C. Fertilization and electroporations were carried out as previously described [24]. After incubation with X-Gal, only well-developed embryos that showed β-galactosidase staining in any tissue were counted. Each construct was electroporated and scored in triplicate, using embryos obtained from different batches of animals collected from the same location.

### Plasmid Construction

All genomic fragments were PCR-amplified from *C. intestinalis* genomic DNA purified from sperm of a single individual and cloned into the pFBΔSP6 vector [24], either directly or after an intermediate cloning step into the vector pGEM-T (Promega). A list of the oligonucleotides employed for PCR amplifications of the initial fragments is provided in Table S1; sequences of the oligonucleotides employed for the construction of the truncations and point mutation constructs are available upon request. The *Ci-ACL* and *Ci-ABCC10* notochord CRMs were identified by screening random genomic regions for *cis*-regulatory activity, essentially as previously described [64].

### Whole-Mount In Situ Hybridization

Digoxigenin-labeled antisense RNA probes were synthesized *in vitro* from existing expressed sequence tags (ESTs) generated and kindly distributed by the Satoh lab [65]. Clone GC29n22 was used for *Ci-Noto8*; GC07k01 for *ABCC10*; GC42d12 for *laminin gamma-1 chain precursor* (aka *laminin B2*; abbreviated as *Ci-lamc1*); GC01e23 for *Ci-FCol1*; GC10b11 for *Ci-Noto5*; GC28e19 for *Ci-ERM*; GC33g19 for *Ci-Noto4* and GC28p14 for *Ci-Noto9*. EST 84P15 (Beckman Coulter Genomics; kindly made available by the Lemaire lab) was used for *Ci-β4GalT*. The *Ci-thbs3* probe was cloned as published previously [25]. Probe labeling and WMISH experiments were performed as previously reported [24].

### Immunohistochemistry and ChIP Assays

Immunohistochemistry was performed on either wild-type or transgenic *C. intestinalis* embryos carrying the *Ci-Bra>GFP* construct [27], essentially as previously described [66], using the published anti-Ci-Bra polyclonal antibody [46]. After an overnight incubation at 4°C with a goat anti-rabbit Alexa Fluor 546 fluorescent antibody (Invitrogen) in PBS, the embryos were washed six times for 10 min in PBS, mounted with Vectashield mounting medium containing DAPI (Vector Laboratories) and imaged using a Leica DMR microscope.

ChIP assays were carried out as previously published [46]. qPCR was performed in triplicate on each of the biological replicates, using SYBR green (USB) in an Applied Biosystems (ABI) Prism 7700 Real-Time qPCR thermocycler. The biological replicates were either two or three in each ChIP-qPCR experiment. To obtain standard curves, duplicates of 5-, 50-, 500-, and 5,000-fold diluted *Ciona* genomic DNA samples were used, starting from 20 ng. The percent input and standard deviation were averaged from immunoprecipitated/input WCE scores. *p*-Values were calculated using a two-tailed Student's *t* test. The negative control was the *18S rRNA* gene (National Center for Biotechnology Information [NCBI] accession number: AJ250778 [http://www.ncbi.nlm.nih.gov]; JGI gene model: gw1.7761.2.1).

## Supporting Information

Figure S1 **Expression patterns of Ci-Bra target genes during early** ***Ciona*** **embryogenesis.** WMISH of *C. intestinalis* embryos fixed at the 110-cell (A,E,I,M,Q), late gastrula (B,F,J,N,R), neural plate/early neurula (C,G,K,O,S), and neurula (D,H,L,P,T) stages. The digoxygenin-labeled antisense RNA probes used are indicated in the left bottom corner of each row. Red arrowheads indicate the regions containing stained notochord cells. (A,E,I,M,Q): vegetal views; all other panels: lateral views, with anterior to the left.
(TIF)

Figure S2 **Position-biased identification of notochord CRMs associated with** ***bona fide*** **Ci-Bra downstream genomic loci.** (A,C,E,G,I,K,M,O,Q,S) Schematic representation of ten genomic loci from which notochord CRMs were isolated. Notochord CRMs are symbolized by red rectangles, and gene names are italicized above the corresponding gene models. Exons are symbolized by rectangles, introns by lines. Dashed lines indicate parts of the coding regions that are not depicted; the last predicted exon is shaped as an arrow pointing towards its 3′-end to indicate the direction of transcription. The Ci-Bra-downstream gene models are colored in dark blue; neighboring gene models are colored in light blue. Gene models are approximate. Intervals that do not contain gene models are abbreviated by parallel diagonal lines. (B,D,F,H,J,L,N,P,R,T) *C. intestinalis* embryos electroporated at the one-cell stage with the CRMs indicated in red on the left side, fixed at the mid-tailbud stage and stained with X-Gal. Anterior is to the left, dorsal is up. Representative cells of stained tissues are indicated by arrowheads, color-coded as follows: red, notochord; blue, CNS; green, epidermis and epidermal neurons; purple, mesenchyme; orange, muscle; yellow, endoderm.
(TIF)

Figure S3 **Activity of additional genomic fragments isolated from Ci-Bra-downstream notochord genes.** (A,C,F,H,J,K,L) Schematic representations of seven loci of Ci-Bra-downstream notochord genes. Gene names are italicized above the corresponding gene models. (B,D,E,G,I) Mid-tailbud *Ciona* embryos electroporated at the one-cell stage with the genomic fragments schematized on the left by colored rectangles, fixed and stained at the late tailbud stage. Grey rectangles indicate inactive genomic fragments; colored rectangles indicate genomic regions displaying *cis*-regulatory activity in tissues other than the notochord. These regions are color-coded as follows: aqua, mixed tissues; violet, epidermis and possibly some regions of the nervous systems; green, epidermis; orange, predominantly muscle and mesenchyme. Arrowheads are color-coded as in Figure S2. In (G) the epidermal cells of both trunk and tail are uniformly stained.
(TIF)

Figure S4 **Detailed analysis of the** ***Ci-Noto4*** **notochord CRM.** Sequence-unbiased truncations and site-directed mutation analysis of the *Ci-Noto4* notochord CRM. “++” and “−” signs are used to show presence or absence of notochord activity, respectively. Binding sites are indicated in the key.
(TIF)

Figure S5 **Structure and truncation analysis of the** ***Ci-Noto8*** **notochord CRM.** (A) Structure of the 0.97-kb notochord CRM associated with the *Ci-Noto8* gene and truncations that were used to identify the minimal sequences required for its activity. Red and grey rectangles symbolize genomic fragments displaying or lacking notochord activity, respectively. All sequences depicted in this figure as “TNNCAC” are listed in Table 1. (B,C) Low-magnification microphotographs of embryos electroporated with (B) the 0.97-kb CRM and (C) the 955-bp truncation depicted in (A). Red arrowheads indicate embryos with notochord staining.
(TIF)

Figure S6 **Detailed mutation analysis of the** ***Ci-lamc1*** **notochord CRM.** (A–E) Low-magnification group microphotographs of embryos from the same batch of animals, electroporated in parallel with constructs containing mutant versions of the 122-bp *Ci-lamc1* notochord CRM (see Figure 5F). Abbreviations: T*n*M, construct carrying a mutation in one of the Ci-Bra binding sites; FoxM, construct carrying a mutation in the putative binding site for a transcription factor of the Fox family. (F) Quantification of the activity of the constructs shown in (A–E) and in Figure 7F in notochord and/or other tissues, plotted as described in Figure 2H. The number of embryos scored (*n*) for each construct is reported below the *x*-axis.
(TIF)

Figure S7 **Expression pattern of the** ***Ci-ABCC10*** **gene during notochord development.** WMISH of wild-type *Ciona* embryos at mid-gastrula (A), late gastrula (B), mid-neurula (C), early tailbud (D), mid-tailbud (E), and late tailbud (F). Stained notochord cells are indicated by a red arrowhead; pink arrowheads indicate weak notochord staining. The embryo in (A) is shown in a vegetal view. The embryo in (B) is oriented with anterior to the left. Embryos in (C–F) are oriented with anterior to the left and dorsal to the top.
(TIF)

Figure S8 **Phylogenetic footprints of the two most conserved notochord CRMs.** Images of the JGI genome browser v2.0 (http://genome.jgi-psf.org/Cioin2/Cioin2.download.ftp.html; [67]) showing the alignment of the *C. intestinalis* minimal notochord CRM sequences (yellow rectangles) to the homologous regions of the *C. savignyi* genome, as provided by the VISTA whole-genome alignment of the two species (http://pipeline.lbl.gov/cgi-bin/gateway2). Conserved non-coding sequences are shown as pink areas. Below each depiction, the detailed sequence alignment is shown; conserved sequences are highlighted in pink, functional Ci-Bra binding sites are indicated in red font and boxed in red. (A) Alignment of the minimal 248-bp *Ci-Noto9* CRM (see Figure 4H–4L), on chromosome 03p [68]. (B) Alignment of the minimal 65-bp *Ci-FCol1* CRM (Figure 3A–3G), on chromosome 07q. A non-conserved Ci-Bra binding site is highlighted in grey. The following parameters were used for the alignments in (A) and (B): calculation window, 100 bp; minimum conservation width, 100 bp; conservation identity, 70%.
(TIF)

Table S1 **Cloning primers used to generate the main CRM constructs, not including restriction sites.**
(DOC)

Table S2 **Distance of functional Ci-Bra binding sites from putative transcription start sites.**
(DOC)

Table S3 **Primers used for qPCR-ChIP analysis.**
(DOC)

## Abbreviations

ChIP: chromatin immunoprecipitation
Ci-Bra: *Ciona* Brachyury
CNS: central nervous system
CRM: *cis*-regulatory module
WMISH: whole-mount *in situ* hybridization
X-Gal: 5-bromo-4-chloro-3-indolyl-β-D-galactopyranoside
