# Supplementary figures and images for: Functional Brachyury Binding Sites Establish a Temporal Read-out of Gene Expression in the Ciona Notochord
Source: PLoS Biol. 2013 Oct 29;11(10):e1001697. doi: 10.1371/journal.pbio.1001697 (PMC3812116; doi:10.1371/journal.pbio.1001697)

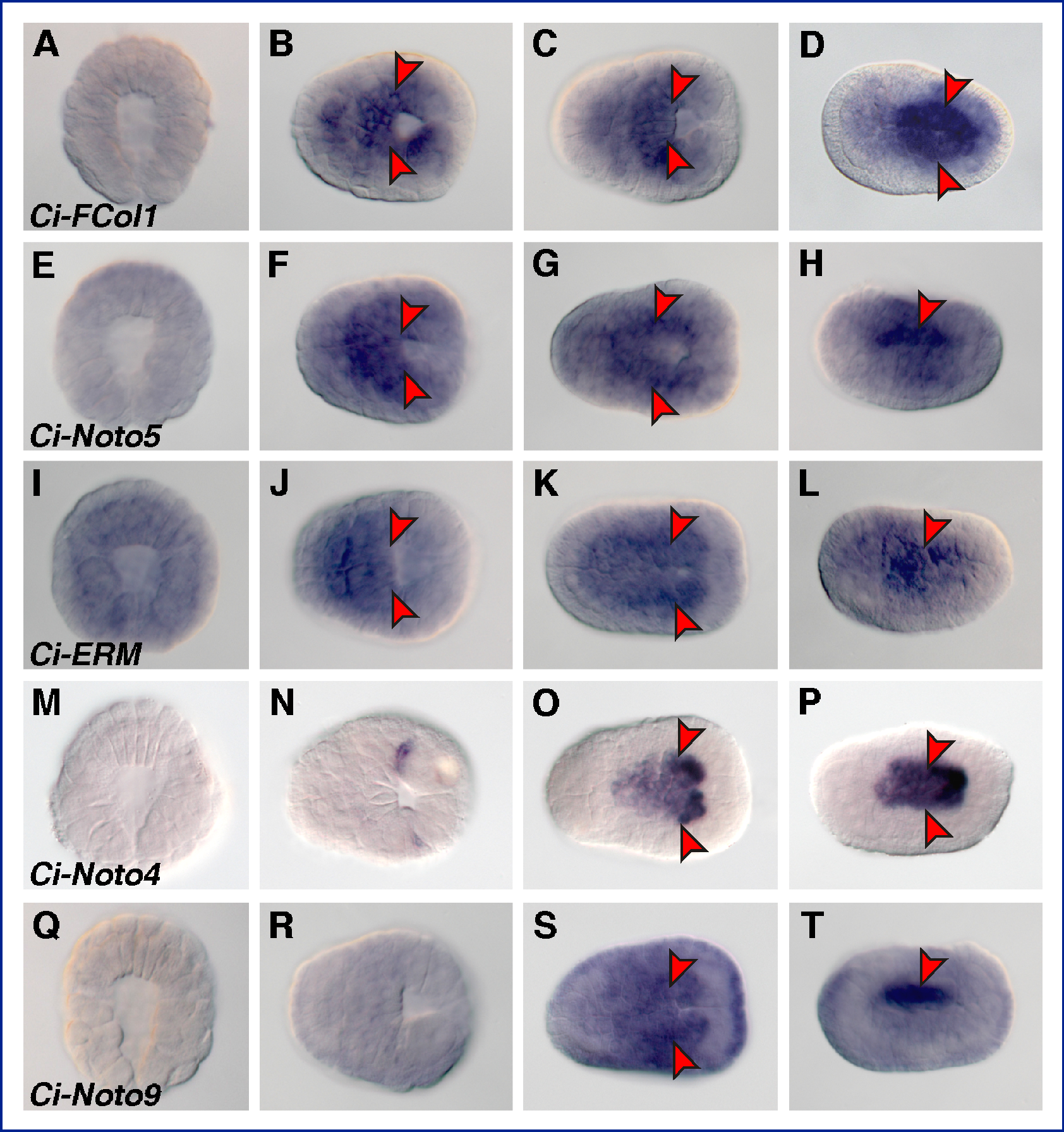

Supplement: Figure S1 — Expression patterns of Ci-Bra target genes during early Ciona embryogenesis. WMISH of C. intestinalis embryos fixed at the 110-cell (A,E,I,M,Q), late gastrula (B,F,J,N,R), neural plate/early neurula (C,G,K,O,S), and neurula (D,H,L,P,T) stages. The digoxygenin-labeled antisense RNA probes used are indicated in the left bottom corner of each row. Red arrowheads indicate the regions containing stained notochord cells. (A,E,I,M,Q): vegetal views; all other panels: lateral views, with anterior to the left. (TIF) [file pbio.1001697.s001.tif]

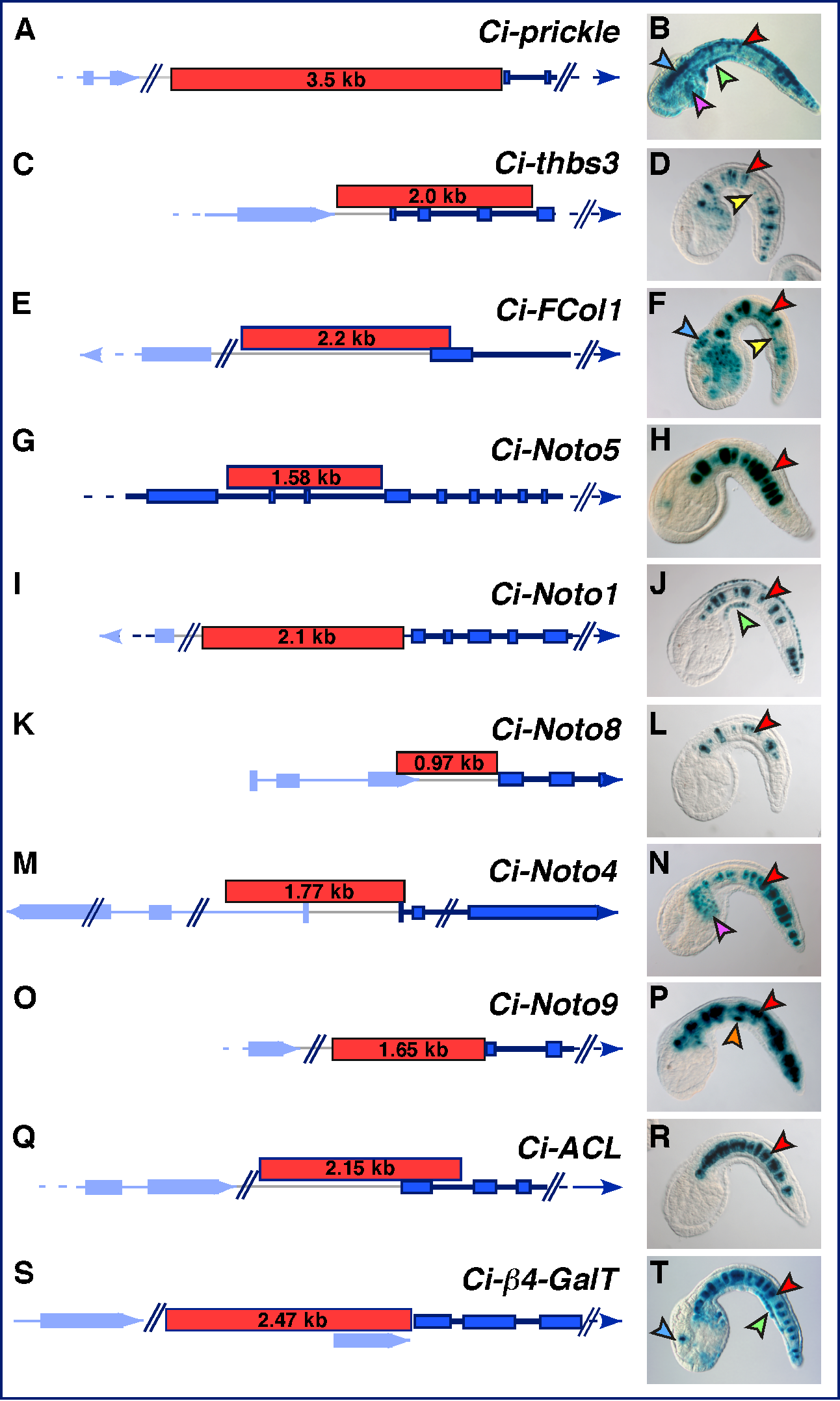

Supplement: Figure S2 — Position-biased identification of notochord CRMs associated with bona fide Ci-Bra downstream genomic loci. (A,C,E,G,I,K,M,O,Q,S) Schematic representation of ten genomic loci from which notochord CRMs were isolated. Notochord CRMs are symbolized by red rectangles, and gene names are italicized above the corresponding gene models. Exons are symbolized by rectangles, introns by lines. Dashed lines indicate parts of the coding regions that are not depicted; the last predicted exon is shaped as an arrow pointing towards its 3′-end to indicate the direction of transcription. The Ci-Bra-downstream gene models are colored in dark blue; neighboring gene models are colored in light blue. Gene models are approximate. Intervals that do not contain gene models are abbreviated by parallel diagonal lines. (B,D,F,H,J,L,N,P,R,T) C. intestinalis embryos electroporated at the one-cell stage with the CRMs indicated in red on the left side, fixed at the mid-tailbud stage and stained with X-Gal. Anterior is to the left, dorsal is up. Representative cells of stained tissues are indicated by arrowheads, color-coded as follows: red, notochord; blue, CNS; green, epidermis and epidermal neurons; purple, mesenchyme; orange, muscle; yellow, endoderm. (TIF) [file pbio.1001697.s002.tif]

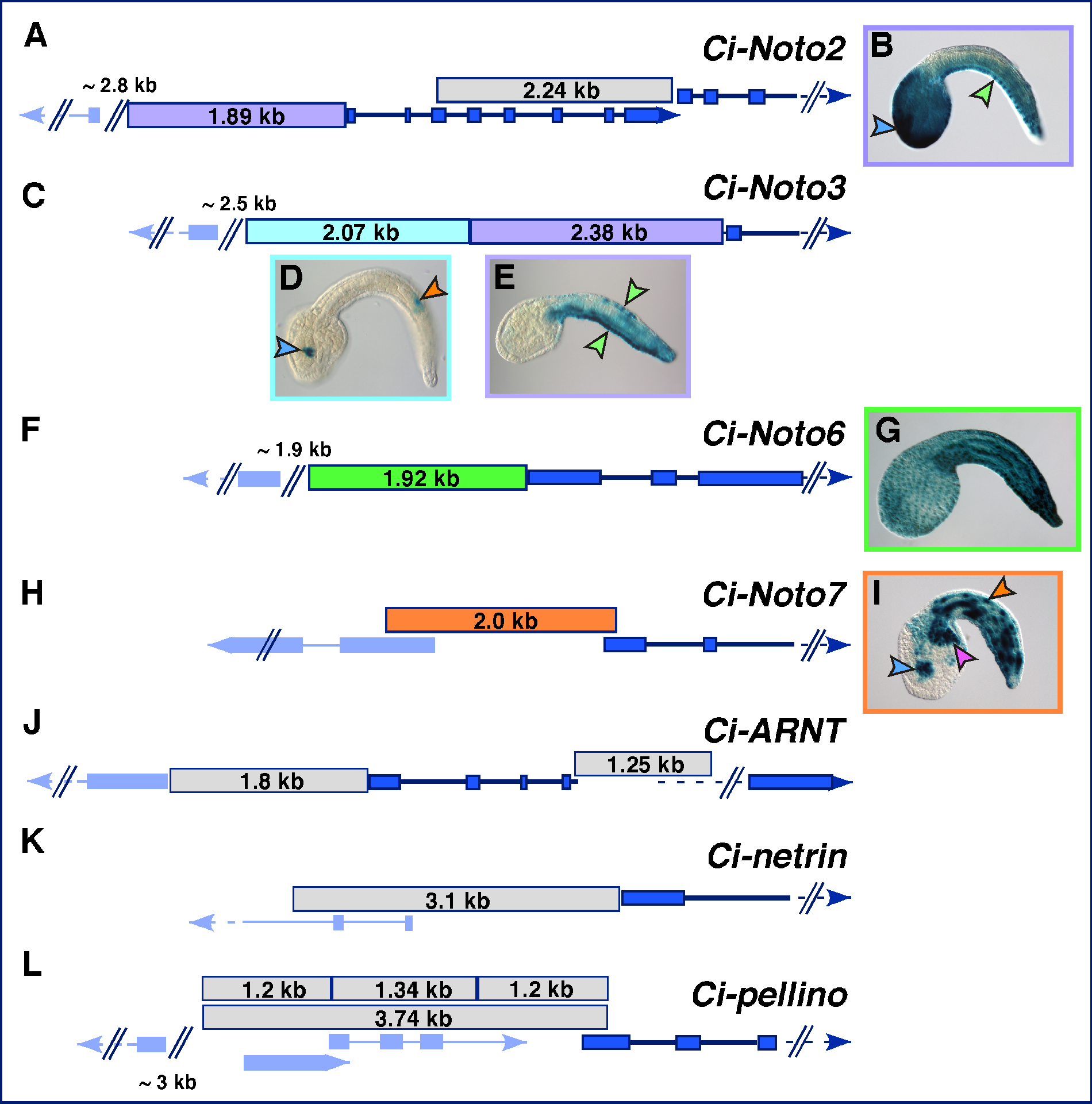

Supplement: Figure S3 — Activity of additional genomic fragments isolated from Ci-Bra-downstream notochord genes. (A,C,F,H,J,K,L) Schematic representations of seven loci of Ci-Bra-downstream notochord genes. Gene names are italicized above the corresponding gene models. (B,D,E,G,I) Mid-tailbud Ciona embryos electroporated at the one-cell stage with the genomic fragments schematized on the left by colored rectangles, fixed and stained at the late tailbud stage. Grey rectangles indicate inactive genomic fragments; colored rectangles indicate genomic regions displaying cis-regulatory activity in tissues other than the notochord. These regions are color-coded as follows: aqua, mixed tissues; violet, epidermis and possibly some regions of the nervous systems; green, epidermis; orange, predominantly muscle and mesenchyme. Arrowheads are color-coded as in Figure S2. In (G) the epidermal cells of both trunk and tail are uniformly stained. (TIF) [file pbio.1001697.s003.tif]

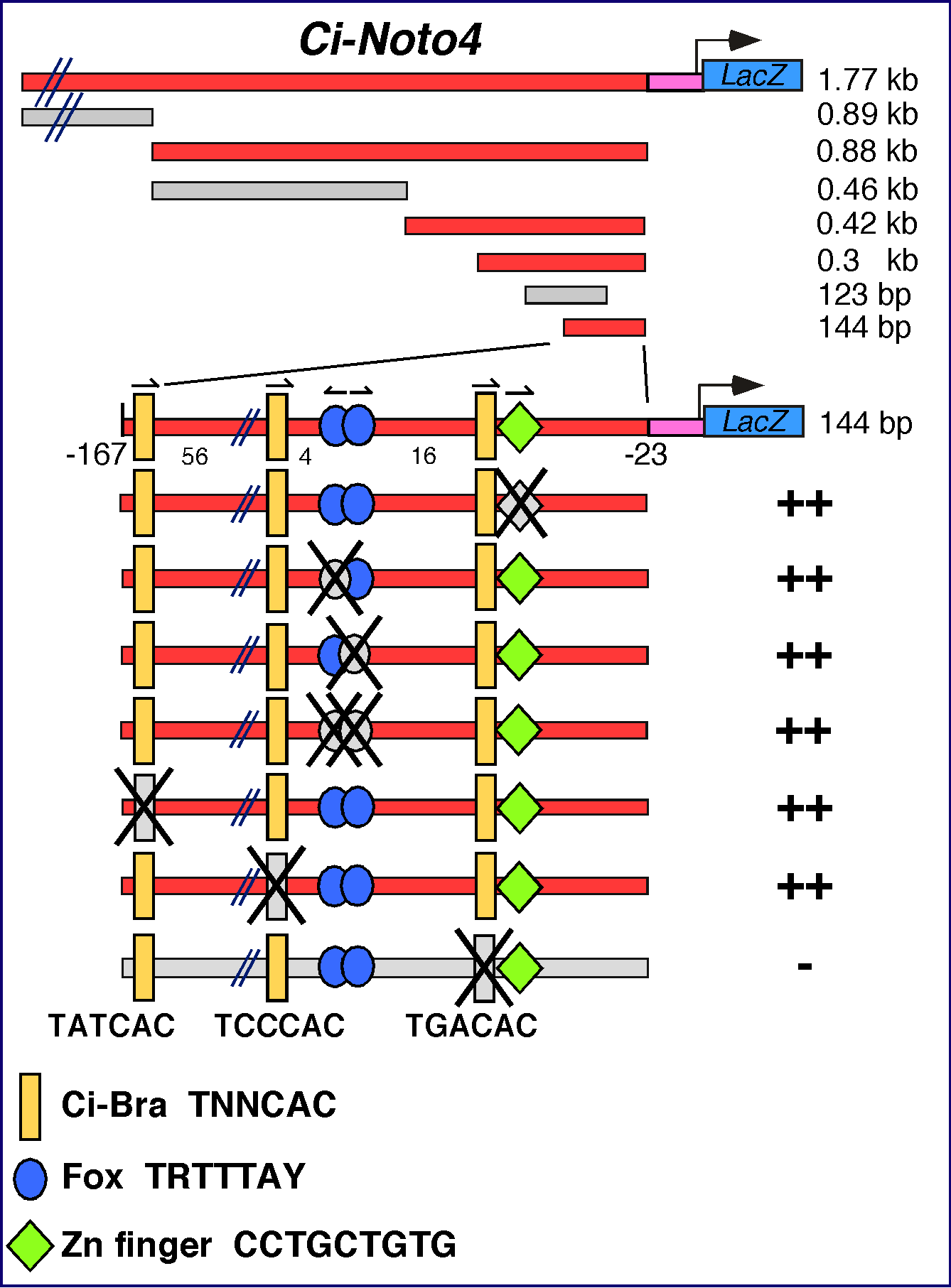

Supplement: Figure S4 — Detailed analysis of the Ci-Noto4 notochord CRM. Sequence-unbiased truncations and site-directed mutation analysis of the Ci-Noto4 notochord CRM. “++” and “−” signs are used to show presence or absence of notochord activity, respectively. Binding sites are indicated in the key. (TIF) [file pbio.1001697.s004.tif]

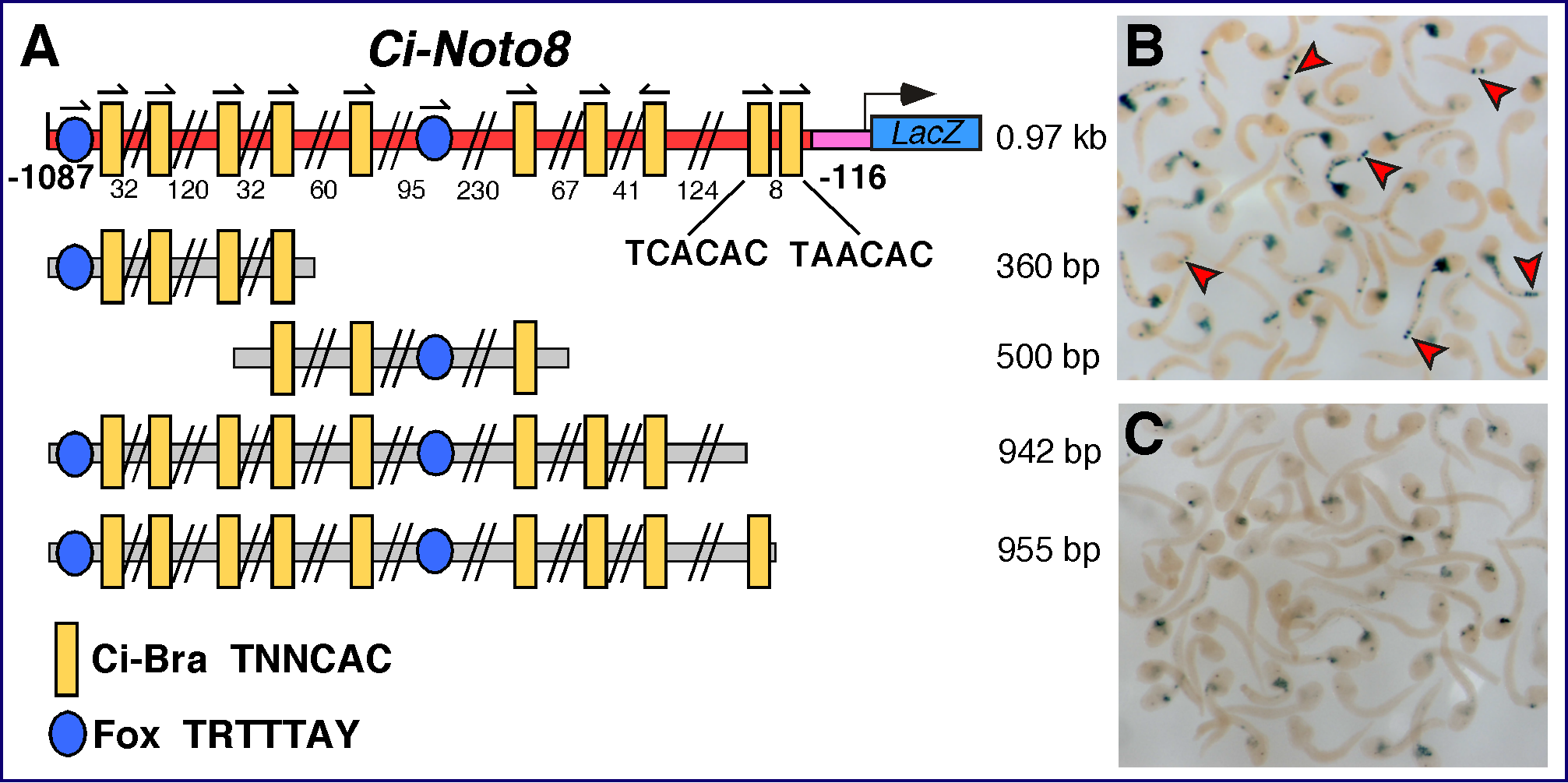

Supplement: Figure S5 — Structure and truncation analysis of the Ci-Noto8 notochord CRM. (A) Structure of the 0.97-kb notochord CRM associated with the Ci-Noto8 gene and truncations that were used to identify the minimal sequences required for its activity. Red and grey rectangles symbolize genomic fragments displaying or lacking notochord activity, respectively. All sequences depicted in this figure as “TNNCAC” are listed in Table 1. (B,C) Low-magnification microphotographs of embryos electroporated with (B) the 0.97-kb CRM and (C) the 955-bp truncation depicted in (A). Red arrowheads indicate embryos with notochord staining. (TIF) [file pbio.1001697.s005.tif]

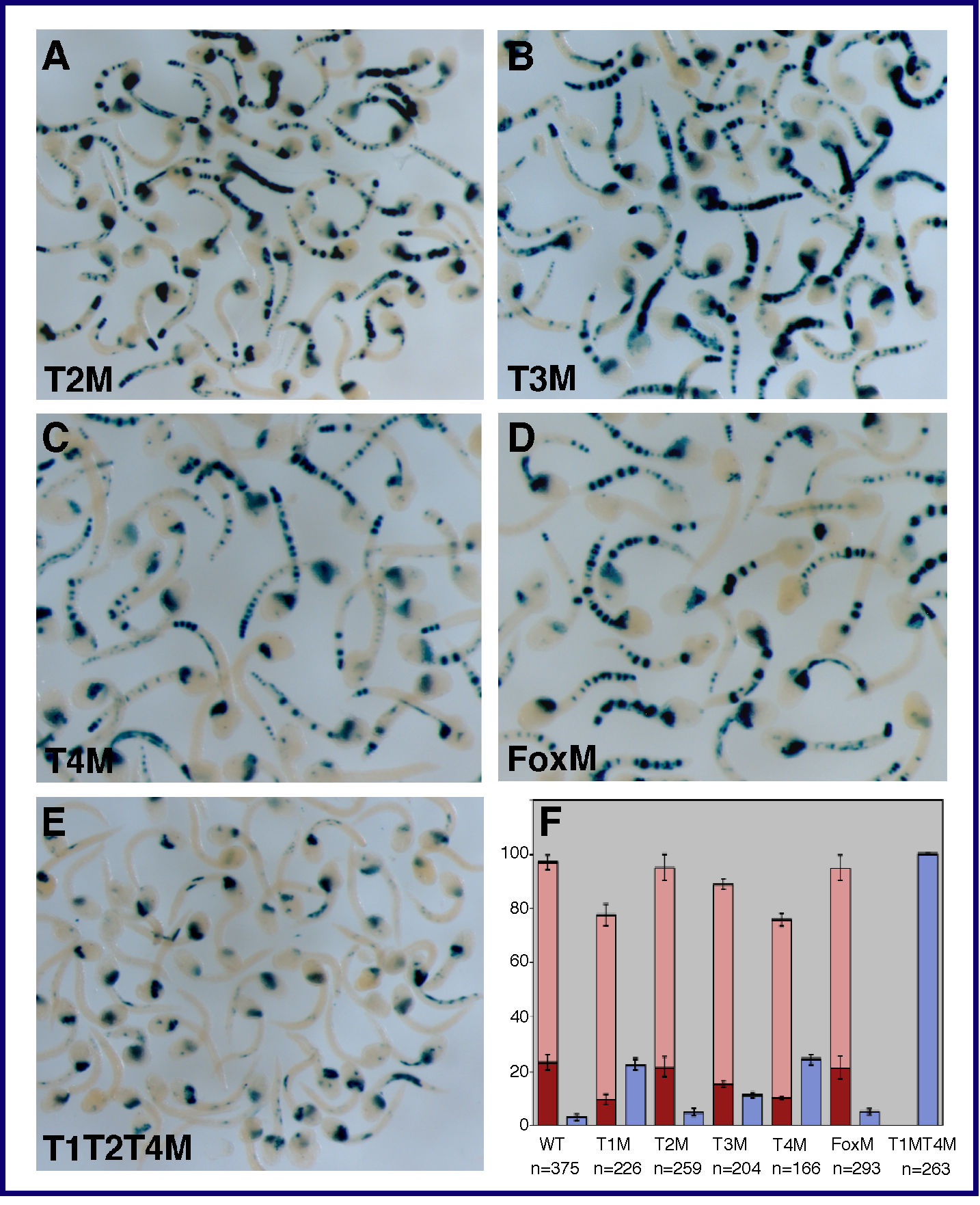

Supplement: Figure S6 — Detailed mutation analysis of the Ci-lamc1 notochord CRM. (A–E) Low-magnification group microphotographs of embryos from the same batch of animals, electroporated in parallel with constructs containing mutant versions of the 122-bp Ci-lamc1 notochord CRM (see Figure 5F). Abbreviations: TnM, construct carrying a mutation in one of the Ci-Bra binding sites; FoxM, construct carrying a mutation in the putative binding site for a transcription factor of the Fox family. (F) Quantification of the activity of the constructs shown in (A–E) and in Figure 7F in notochord and/or other tissues, plotted as described in Figure 2H. The number of embryos scored (n) for each construct is reported below the x-axis. (TIF) [file pbio.1001697.s006.tif]

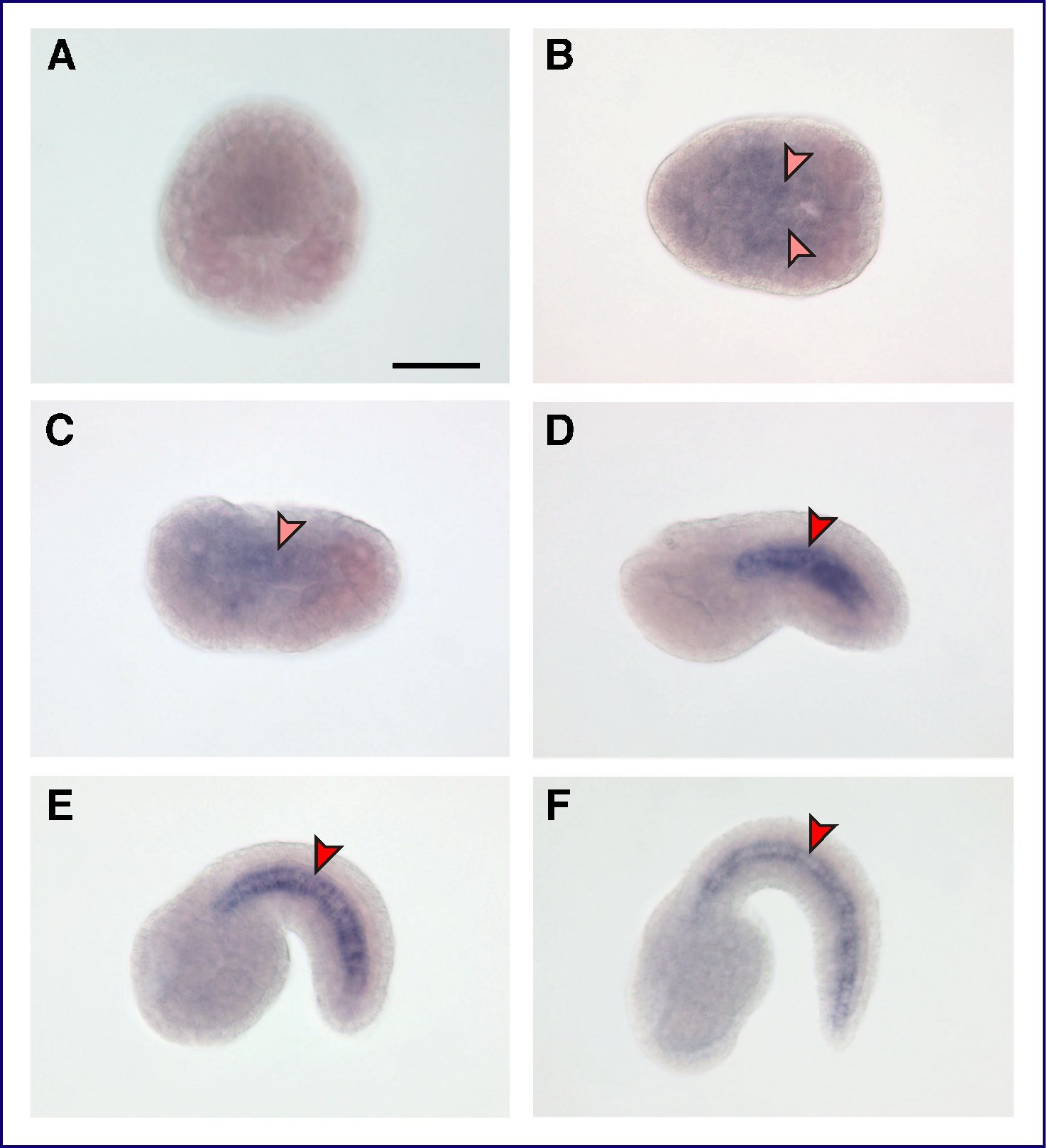

Supplement: Figure S7 — Expression pattern of the Ci-ABCC10 gene during notochord development. WMISH of wild-type Ciona embryos at mid-gastrula (A), late gastrula (B), mid-neurula (C), early tailbud (D), mid-tailbud (E), and late tailbud (F). Stained notochord cells are indicated by a red arrowhead; pink arrowheads indicate weak notochord staining. The embryo in (A) is shown in a vegetal view. The embryo in (B) is oriented with anterior to the left. Embryos in (C–F) are oriented with anterior to the left and dorsal to the top. (TIF) [file pbio.1001697.s007.tif]

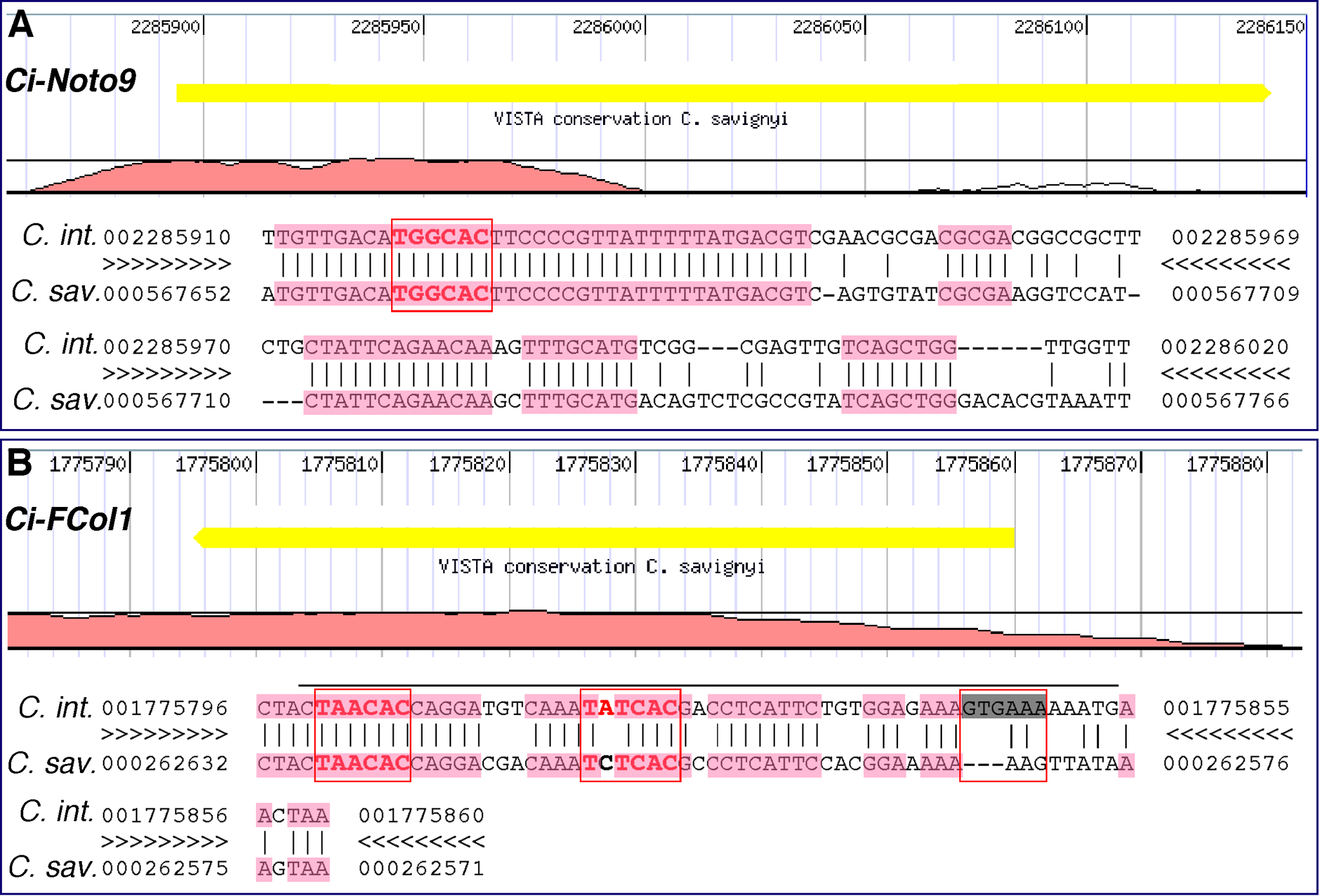

Supplement: Figure S8 — Phylogenetic footprints of the two most conserved notochord CRMs. Images of the JGI genome browser v2.0 (http://genome.jgi-psf.org/Cioin2/Cioin2.download.ftp.html; [67]) showing the alignment of the C. intestinalis minimal notochord CRM sequences (yellow rectangles) to the homologous regions of the C. savignyi genome, as provided by the VISTA whole-genome alignment of the two species (http://pipeline.lbl.gov/cgi-bin/gateway2). Conserved non-coding sequences are shown as pink areas. Below each depiction, the detailed sequence alignment is shown; conserved sequences are highlighted in pink, functional Ci-Bra binding sites are indicated in red font and boxed in red. (A) Alignment of the minimal 248-bp Ci-Noto9 CRM (see Figure 4H–4L), on chromosome 03p [68]. (B) Alignment of the minimal 65-bp Ci-FCol1 CRM (Figure 3A–3G), on chromosome 07q. A non-conserved Ci-Bra binding site is highlighted in grey. The following parameters were used for the alignments in (A) and (B): calculation window, 100 bp; minimum conservation width, 100 bp; conservation identity, 70%. (TIF) [file pbio.1001697.s008.tif]
