# Supplementary material for: Functional Brachyury Binding Sites Establish a Temporal Read-out of Gene Expression in the Ciona Notochord
Source: PLoS Biol. 2013 Oct 29;11(10):e1001697. doi: 10.1371/journal.pbio.1001697 (PMC3812116; doi:10.1371/journal.pbio.1001697)
Supplement: Table S1 — Cloning primers used to generate the main CRM constructs, not including restriction sites. (DOC) [file pbio.1001697.s009.doc]

| **Table S1: Cloning primers used to generate the main CRM constructs, not including restriction sites.** | | |
| --- | --- | --- |
| **CRM construct** | **Forward primer sequence (5’-3’)** | **Reverse primer sequence (5’-3’)** |
| *Ci-Noto1* 2.1 kb | TTCGCATGATAGACAACCATGGTCGACGC | ACATCAGTTAAATCAAGTTCTTAATACACTGTC |
| *Ci-Noto4* 1.77 kb | GATACATGGCTAGACAGAACTTAGTAGC | GAAATAAGATTCTGCTTGACCTCCATTG |
| *Ci-Noto5* 1.58 kb | AATACAAAAGTGAATTATGCAGCAACGTC | ACTGGATGCTGTTTGATCCTGGAG |
| *Ci-Noto8* 1.0 kb | CAATTAATGGAGACACGA | ATCCGCAACATTCCACTG |
| *Ci-Noto9* 1.65 kb | CCGGGTATGTAGGTCTTGT | CATAATTTCCATGTTTAAGTTTACTTG |
| *Ci-β4-galT* 2.4 kb | TATGGATCACACATATGTTGCAATTCC | AACAGTTATACGAAACAGGCGATACC |
| *Ci-FCol1* 2.2 kb | CCTTTGAACCCTCCTGCTCG | GGCTAGAACGAGCGAAAAAGTC |
| *Ci-prickle* 3.5 kb | ATAGCTTTGGATTGACTAAGACGTATAG | AAGGCAGCCGCGGTTGATAACTTAATCC |
| *Ci-thbs3* 2.0 kb | GCGAACCACTGATTAAACGTTTTGG | ACTAAAGAAACGAAGTTGGTAAATAAA |
| *Ci-Noto2* 2.24 kb | AGCGCAGCTGAAAGGTTAATAGTTCGCAG | CGGAAATCACCGTTACAATCGCCTTATG |
| *Ci-Noto2* 1.89 kb | GTGAGGAATCCTTTACGTCGT | TGCGCTGTTTCTCTATGAT |
| *Ci-Noto3* 2.38 kb | GTGCAAGAAATGTAATCAGTATATGC | CATTTAAGTCCGAATATGGCGCCTTGGAAC |
| *Ci-Noto3* 2.07 kb | GTTACTTGCTACAGATCGTTGAAGGCTCCAC | CTAATATCTCTAACGTTATTGCCGATTGAGC |
| *Ci-Noto6* 1.92 kb | AATTTGAATGTTCTATATGTT | CCTTTAAAACAACCTAGCATA |
| *Ci-Noto7* 2.0 kb | ATTTCATTGCAAGTACCATTA | GTATCAGGCAGCGTAAAAC |
| *Ci-ARNT* 1.8 kb | TAGTAGGATGGGGGAAGATGG | AGGACTTGTATTGGAAACAGTATCTG |
| *Ci-ARNT* 1.25 kb | TGGCTACGAACGAGTTCATTTGC | ATCGTTCAGGTACAGGTTGTTGG |
| *Ci-netrin* 3.1 kb | CTTACCCAACAGTACTTTATAC | GTGGGTCCTGCATGGATACAC |
| *Ci-pellino* 3.74 kb | TCCCGCAAGTGCTGTGTAACAGCTG | GCCAAGAGTCTTCTAAGTAACC |
| *Ci-pellino* 1.2 kb | GCCAAGAGTCTTCTAAGTAACCG | ACAATCCCTTTATGCTCGTCGGCT |
| *Ci-pellino* 1.34 kb | GTAAGCCGACGAGCATAAAGGGA | TGGTAACCTGTAAGCAGGCATGA |
| *Ci-pellino* 1.2 kb | AGCAATAACAGTTTATAACAACTG | CGCAAGTGCTGTGTAACAGCTGTA |
| *Ci-ERM*  560 bp | GATTTAATGTTCGATGTTAACGTTTTACC | ATACACATTTACAACGTAACCGTT |
| *Ci-ABCC10*  772 bp | GAGTAAAGTCGCAAACACATATAGCA | TGAAGGGGGTTAAAATGACTTCATTTG |
| *Ci-lamc1*  270 bp | GTTATAATGGCACAAAGACGC | GCAGTCACTTAGCAATGATGTC |
| *Ci-Noto1* on endogenous promoter  600 bp | TGATTTAACTGATGTGGAACTCCTTTAG | CATTTTAAATGAACGTATTTGATTGGGT |
| *Ci-FCol1* on endogenous promoter  2.986 kb | GTTGTTAATTGCAGCACCGTCGGTCCT | TTTAAAAGAGCGGCGGGCAGTCACGC |
| *Ci-lamc1* on endogenous promoter  862 bp | ATAATGGCACAAAGACGCTATTGCTA | TCGAAACAGATGCCTAAACGTCATCCTG |
| *Ci-ABCC10* on endogenous promoter  2.272 kb | TGTGGATTATTTAATACAAGATTTTAAGTTG | ATCCTCTACTGTGACATCACAATACCCAT |
| *Ci-ERM* on endogenous promoter  2.44 kb | TTTCGTAAGGTTATAGTTAGGTTGTTTAG | TATTACATCACAGTAAGGACAATATTATC |
| *Ci-β4-galT* on endogenous promoter  365 bp | CAATGTTGCTCTTACGTGTATATGCTGGTG | CAATAAAATAAACAGTTAAACCGGTTAAC |
