# Supplementary material for: Functional Brachyury Binding Sites Establish a Temporal Read-out of Gene Expression in the Ciona Notochord
Source: PLoS Biol. 2013 Oct 29;11(10):e1001697. doi: 10.1371/journal.pbio.1001697 (PMC3812116; doi:10.1371/journal.pbio.1001697)
Supplement: Table S2 — Distance of functional Ci-Bra binding sites from putative transcription start sites. (DOC) [file pbio.1001697.s010.doc]

| **Table S2.** **Distance of functional Ci-Bra binding sites from putative transcription start sites** | | |
| --- | --- | --- |
| **Ci-Bra binding site** | **Location** | **Gene model used as a reference** |
| *Ci-Noto1* TGGCAC | -558 bp | KH.L20.18.v1.B.SL2-1 |
| *Ci-Noto4* TGACAC | -59 bp | KH.L18.30.v1.A.nonSL1-1 |
| *Ci-Noto8* TAACAC | -390 bp | KH.C11.665.v1.A.ND1-1 |
| *Ci-Noto9* TGGCAC | -363 bp | KH.L13.3.v1.A.SL2-1 |
| *Ci-ABCC10* TAACAC | +2301 bp | KH.C10.36.v1.A.ND1-1 |
| *Ci-FCol1* TATCAC | -259 bp | KH.C7.633.v1.A.ND1-1 |
| *Ci-FCol1* TAACAC | -241 bp | KH.C7.633.v1.A.ND1-1 |
| *Ci-Noto5* TAACAC | +9192 bp | KH.L153.32.v1.A.ND1-1 |
| *Ci-Noto5* TCACAC | +9229 bp | KH.L153.32.v1.A.ND1-1 |
| *Ci-ERM* TAACAC | -2595 bp | KH.C12.129.v1.A.SL3-1 |
| *Ci-ERM* TCACAC | -2554 bp | KH.C12.129.v1.A.SL3-1 |
| *Ci-lamc1* TCACAC | -771 bp | KH.C7.167.v1.A.SL3-1 |
| *Ci-lamc1* TCGCAC | -676 bp | KH.C7.167.v1.A.SL3-1 |
| *Ci-thbs3* TCGCAC | -462 bp | KH.C6.164.v1.A.SL1-1 |
| *Ci-thbs3* TAACAC | -460 bp | KH.C6.164.v1.A.SL1-1 |
| *Ci-thbs3* TCGCAC | -388 bp | KH.C6.164.v1.A.SL1-1 |
