# Supplementary material for: Functional Brachyury Binding Sites Establish a Temporal Read-out of Gene Expression in the Ciona Notochord
Source: PLoS Biol. 2013 Oct 29;11(10):e1001697. doi: 10.1371/journal.pbio.1001697 (PMC3812116; doi:10.1371/journal.pbio.1001697)
Supplement: Table S3 — Primers used for qPCR-ChIP analysis. (DOC) [file pbio.1001697.s011.doc]

| **Table S3.** **Primers used for qPCR-ChIP analysis.** | | |
| --- | --- | --- |
| **CRM** | **Forward primer sequence (5’-3’)** | **Reverse primer sequence (5’-3’)** |
| *Ci-tune* | GTGTTGCGTACACACTCAAAGTCAG | GCAGGGCAGTTCTGATAAACACGTTGT |
| *Ci-Noto1* | CGCTGGGTGTGGTGAAACAATGGCT | CAGAAAGCGTTTTCGCGTTCATGCCTC |
| *Ci-Noto4* | TCGGGGCGATGAAAGAGTGTTTGC | AAGGCGACACCAACAAGGCGTCCATT |
| *Ci-Noto5* | GCATGAATAACAGTTTGCCGAAAGTAACAC | AGAGCGCTGTTCTAGTTAAAATTACAGCACTG |
| *Ci-Noto9* | cacgataccgatctttcctttttggg | TTGTTCTGAATAGCAGAAGCGGCCGT |
| *Ci-FCol1* | CCGGTGTGTTTCTGTATACTGACAGC | AGGGGCGGAGTGCGAAATGCAAAG |
| *Ci-lamc1* | GACAAGAACGTCCGCATTCTCACATCG | CCAGACTAAGTCCCGCCACACGATAT |
| *Ci-ACL* | GCCTTTGTAATGTAGGCTATGCAAGCG | CAAGCACAGTACTAGTTCTGTTTACAGAAGC |
| *Ci-18S rRNA* | CGAAGACGATCAGATACCGTCCTAG | TGGTGGTGCCCTTCCGTCAATTCC |
